# Characterization of the Apelin/Elabela Receptors (APLNR) in Chickens, Turtles, and Zebrafish: Identification of a Novel Apelin-Specific Receptor in Teleosts

**DOI:** 10.3389/fendo.2018.00756

**Published:** 2018-12-13

**Authors:** Jiannan Zhang, Yawei Zhou, Chenlei Wu, Yiping Wan, Chao Fang, Jing Li, Wenqian Fang, Ran Yi, Guoqiang Zhu, Juan Li, Yajun Wang

**Affiliations:** Key Laboratory of Bio-Resources and Eco-Environment of Ministry of Education, College of Life Sciences Sichuan University, Chengdu, China

**Keywords:** chicken, turtle, zebrafish, Apelin, Elabela, APLNR

## Abstract

Apelin receptor(s) (APLNR) are suggested to mediate the actions of apelin and Elabela (ELA) peptides in many physiological processes, including cardiovascular development and food intake in vertebrates. However, the functionality of APLNR has not been examined in most vertebrate groups. Here, we characterized two APLNRs APLNR1, APLNR2) in chickens and red-eared sliders, and three APLNRs in zebrafish (APLNR2a, APLNR2b, APLNR3a), which are homologous to human APLNR. Using luciferase-reporter assays or Western blot, we demonstrated that in chickens, APLNR1 (not APLNR2) expressed in HEK293 cells was potently activated by chicken apelin-36 and ELA-32 and coupled to Gi-cAMP and MAPK/ERK signaling pathways, indicating a crucial role of APLNR1 in mediating apelin/ELA actions; in red-eared sliders, APLNR2 (not APLNR1) was potently activated by apelin-36/ELA-32, suggesting that APLNR2 may mediate apelin/ELA actions; in zebrafish, both APLNR2a and APLNR2b were potently activated by apelin-36/ELA-32 and coupled to Gi-cAMP signaling pathway, as previously proposed, whereas the novel APLNR3a was specifically and potently activated by apelin. Similarly, an apelin-specific receptor (APLNR3b) sharing 57% sequence identity with zebrafish APLNR3a was identified in Nile tilapia. Collectively, our data facilitates the uncovering of the roles of APLNR signaling in different vertebrate groups and suggests a key functional switch between APLNR1 and APLNR2/3 in mediating the actions of ELA and apelin during vertebrate evolution.

## Introduction

Apelin receptor (APLNR) has been identified as a class A (rhodopsin-like) G protein-coupled receptor (GPCR) and originally named APJ, which shares a relatively high amino acid sequence identity (31%) with angiotensin II receptor (AT1), but displays no specific binding toward angiotensin II peptide (1, 2). A later study revealed that APLNR can bind a novel peptide of 36 amino acids, “apelin-36,” which was first extracted from bovine stomach (3). In addition to apelin-36, multiple short forms of apelin peptide with an identical C-terminus, such as apelin-17, apelin-13, and pyroglutamyl form of apelin-13 (pGlu-apelin-13), have been identified in mammals (2, 4–6). All these peptides are derived from a large preproapelin precursor encoded by the apelin (*APLN*) gene, and have been shown to be capable of binding APLNR with high affinities (3, 6). It is reported that APLNR is capable of coupling to Gi protein(s) and its activation can modulate multiple signaling pathways, including the inhibition of cAMP signaling pathway and activation of ERK signaling cascades (4–8). Since the discovery of this ligand-receptor pair in 1998 (3), there has been increasing evidence showing that APLN-APLNR is widely expressed in mammalian tissues including the brain and heart (7). For instances, in rats, both APLN and APLNR are reported to be expressed in various brain regions, with a high expression level noted in the supraoptic nucleus (SON) and the paraventricular nucleus (PVN) of hypothalamus (9–11). In humans, APLNR is expressed in the vascular endothelial and smooth muscle cells and ventricular cardiomyocytes of the heart, while APLN is expressed in the vascular and endocardial endothelial cells (12, 13). Moreover, APLN-APLNR is also expressed in the endothelial cells and/or smooth muscle cells of the vasculature of other human tissues, including the pulmonary vessels, small intrarenal vessels, and the surrounded arteries of adrenal glands (12–14). The wide tissue expression of APLN-APLNR suggests their involvement in the regulation of many physiological processes, such as cardiovascular development and function (15, 16), blood pressure (17, 18), fluid homeostasis (19), angiogenesis (20), neuroendocrine activity (21), heart development (22–24), food intake (25, 26), drinking behavior (27, 28), pituitary hormone secretion (27, 29), metabolism (30), and neuroprotection (31) in the central nervous system (CNS) and peripheral tissues, although some of these findings are contradictory (5). Similar to these mammalian findings, *APLN*/*APLNR* knockdown in zebrafish or *Xenopus laevis* causes abnormalities in vascular development (32, 33), myocardial cell specification and heart formation (24, 34, 35), further highlighting the ancient and conserved roles of *APLN*-APLNR signaling in the regulation of cardiovascular development in vertebrates (36).

Recently, a novel ligand for APLNR named Elabela (ELA) (also called “Toddler” or “Apela”) has been identified in zebrafish (37, 38). Zebrafish ELA has a temporal expression pattern distinct from that of apelin during embryogenesis, and likely acts as an early developmental signal to regulate the movement of mesoendodermal cells, heart development, and angioblast migration (37–39). ELA is a 32-amino acid peptide conserved in vertebrates (37, 38). Despite the little homology shared between ELA and apelin peptides, *in vitro* studies support that ELA can bind to the two APLNRs (APLNRa and APLNRb) in zebrafish and cause receptor internalization (37, 38). Moreover, *in vivo* data show that *APLNR* mutants are phenotypically indistinguishable from *ELA* null zebrafish embryos, both displaying abnormalities in cardiovascular development (37–39). All these findings tend to support the notion that ELA is a potential alternative ligand for APLNRs in zebrafish, though this idea awaits further verification. Consistent with the findings in zebrafish, ELA has been recently shown to be capable of binding and activating APLNR in mammals (40–42). Like apelin, ELA is also suggested to regulate water intake and diuresis in rats (40) and angiogenesis in cultured human umbilical vascular endothelial cells (41). Loss of *ELA* in mice causes the defects in early mesodermal derivatives, cardiovascular malformations, and embryonic lethality (<50% incidence) (43, 44). Interestingly, in human embryonic stem cells (hESC), *ELA* is abundantly expressed and sustains hESC self-renewal via an unknown receptor coupled to PI3K/Akt pathway (45).

Despite the identification of APLNR(s) and its two ligands (i.e., apelin and ELA) in mammals and zebrafish, the structure, functionality and tissue expression of the APLNRs in many vertebrate groups including birds have not been fully characterized. Therefore, in our present study, we aimed to characterize APLNR in several representative vertebrate species including chickens, turtles and zebrafish. Strikingly, we identified two APLNRs (APLNR1 and APLNR2) in chickens and turtles, and three functional APLNRs in zebrafish, including a novel apelin-specific receptor (APLNR3), which has not been reported in any vertebrate before. Undoubtedly, our study establishes a molecular basis to elucidate the roles of APLNRs and their ligands in different vertebrate groups.

## Materials and Methods

### Peptides, Antibodies, Primers, and Chemicals

Chicken apelin-36, apelin-13, ELA32a (with an intra-molecular disulfide bond), ELA32b (without a disulfide bond), and ELA-11, and zebrafish apelin-36 and ELA-36/ELA-22 (without a disulfide bond) peptides were synthesized (>95% purity) by GL Biochem (Shanghai, China) and their structures were verified by mass spectrometry. Restriction enzymes and *Taq* DNA polymerase were purchased from Takara (Dalian, China). Primers used in this study were synthesized by TSINGKE (Beijing, China) and listed in Supplementary Table 2. Monoclonal antibodies for β-actin and ERK1/2 were purchased from Cell Signaling Technology Inc., (CST, Beverly, MA, USA). All chemicals and reagents were purchased from Sigma Aldrich (Shanghai, China) unless stated otherwise.

### Animal Experiments

Adult chickens (Lohmann layers, *Gallus gallus*), zebrafish (*Danio rerio*), Nile tilapia (*Oreochromis niloticus*), and red-eared sliders (*Trachemys scripta elegans*) purchased from local suppliers were sacrificed and various tissues were collected. Zebrafish embryos at consecutive developmental stages (0, 2, 4, 6, 8, 10, 12, 24, and 48 hpf) were also collected. All these animal experiments were conducted in accordance with the Guidelines for Experimental Animals issued by the Ministry of Science and Technology of People's Republic of China. The experimental protocol used in this study was approved by the Animal Ethics Committee of College of Life Sciences, Sichuan University (Chengdu, China).

### Total RNA Extraction, RT-PCR, and Quantitative Real-Time PCR (qPCR)

Total RNA was extracted from animal tissues with RNAzol Reagent (Molecular Research Center, Cincinnati, OH, USA) and dissolved in DEPC-treated H_2_O. Reverse transcription (RT) was performed using MMLV Reverse Transcriptase (Takara, Dalian, China) from 2 μg total RNA. These RT samples were then used in PCR amplification of target genes, or subjected to quantitative real-time PCR assay (qPCR) of gene expression in chicken or zebrafish tissues on the CFX96 Real-time PCR Detection System (Bio-Rad, Hercules, CA, USA), as described in our recent studies (46, 47).

### Rapid Amplification of 5′-cDNA and 3′ cDNA Ends (RACE)

To determine the gene structure of chicken *APLN* and *ELA*, rapid amplification of 5′- or 3′-cDNA ends (RACE) was performed to amplify cDNAs of both genes from brain tissue using SMART-RACE cDNA amplification Kit (Clontech, Palo Alto, CA, USA) according to the manufacturer's instruction. The amplified PCR products were cloned into pTA2 vector (Toyobo, Osaka, Japan) and sequenced (BGI, Beijing, China). The gene structure of *APLN* and *ELA* was then determined by comparing their cDNA sequences with genomic sequences from the chicken genome database (www.ensembl.org/gallus_gallus).

### Identification of *APLNR* Genes in Chickens and Other Vertebrates

Using human *APLNR* cDNA (NM_005161) as a reference, we search its homologous gene(s) in the genome of several representative vertebrate species, including chickens, turtles, *Xenopus tropicalis*, zebrafish, Nile tilapia, spotted gars, *tetraodons, takifugu rubripes* (http://www.ensembl.org). The amino acid sequences of *APLNR(s)* were either predicted according to their genomic sequences or retrieved from GenBank. A phylogenetic tree of APLNRs was constructed using the Neighbor-Joining Method supplied in MEGA6 software (http://www.megasoftware.net).

### Construction of the Expression Plasmid of APLNR(s) From Humans, Chickens, Red-Ear Slider, and Zebrafish

Primers were designed based on sequence information of *APLNRs* from chicken (or western painted turtle, zebrafish, Nile tilapia) genome database (http://www.ensembl.org/). Then the open reading frame (ORF) of each *APLNR* was amplified from the animal [chicken, red-eared slider (*Trachemys scripta elegans*), zebrafish, tilapia] heart tissue with the use of high-fidelity KOD DNA polymerase (Toyobo, Osaka, Japan). The PCR products were cloned into pcDNA3.1 (+) expression vector (Invitrogen, Carlsbad, CA, USA) and sequenced (BGI, Beijing, China). Using genomic DNA extracted from human embryonic kidney 293 (HEK293) cells as the template, human *APLNR* ORF was also amplified by PCR and cloned into pcDNA3.1 (+) expression vector.

### Cell Culture

Human embryonic kidney 293 (HEK293) cells were maintained in DMEM supplemented with 10% (vol/vol) fetal bovine serum (HyClone, Logan, UT, USA), 100 U/ml penicillin G and 100 μg/ml streptomycin (Life Technologies, Grand Island, NY, USA) in a 90-cm culture dish (Nunc, Rochester, NY, USA). Cells were cultured at 37°C in a humidified atmosphere with 5% CO_2_ and routinely sub-cultured every 3 days.

### Functional Characterization of APLNRs in Cultured HEK293 Cells

According to our previously established method, the functionality of APLNRs from chickens, red-eared sliders, zebrafish, Nile tilapia and humans was examined in HEK293 cells by a pGL3-CRE-luciferase reporter or pGL4-SRE-luciferase reporter system, which have been shown to be capable of monitoring receptor-activated (-inhibited) cAMP or MAPK signaling pathways (48–51). In brief, HEK293 cells were cultured on a six-well plate and grown for 24 h before transfection. The cells were then transfected with a mixture containing 700 ng pGL3-CRE-Luciferase reporter construct (or pGL4-SRE-Luciferase reporter construct), 200 ng of receptor expression plasmid [or empty pcDNA3.1(+) vector as a negative control], and 2 μl jetPRIME (Polyplus Transfection, Illkirch, France) in 200 μl buffer. Twenty-four hour later, HEK293 cells were sub-cultured into a 96-well plate at 37°C for an additional 24 h before treatment. After removal of medium from the 96-well plate, the cells were treated with 100 μl peptide-containing medium (or peptide-free medium) for 6 h in the presence or absence of forskolin (5 μM). Finally, HEK293 cells were lysed with 1 × passive lysis buffer for luciferase assay (Promega, Madison, WI, USA) and the luciferase activity of the cell lysate was measured by a Multimode microplate Reader (TriStar LB 941, EG&G Berthold, Germany) according to the manufacturer's instruction.

### Western Blot

HEK293 cells cultured in a 60-mm culture dish (Nunc, Rochester, NY, USA) with 70% confluence were transfected by 1 μg of receptor expression plasmid using jetPRIME reagent according to manufacturers' instruction (Polyplus Transfection, Illkirch, France). After the 24-h transfection, HEK293 cells were sub-cultured into a 24-well plate, serum-deprived for 12 h and then treated by peptides for 10 min. These cells were washed with cold PBS and lysed by RIPA Lysis Buffer (Roche, Basel, Switzerland) on ice. Western blot was used to examine ERK1/2 phosphorylation and β-actin levels in cell lysates.

### Data Analysis

The luciferase activity in each treatment group was expressed as the relative fold changes as compared with their respective control groups. The data were analyzed by non-linear regression followed by one-way ANOVA using GraphPad Prism 5 (GraphPad, San Diego, CA, USA). To validate the results, all experiments were repeated at least twice.

## Results

### Cloning the Full-Length cDNAs of Chicken *APLN* and *ELA*

**Figure 1:**
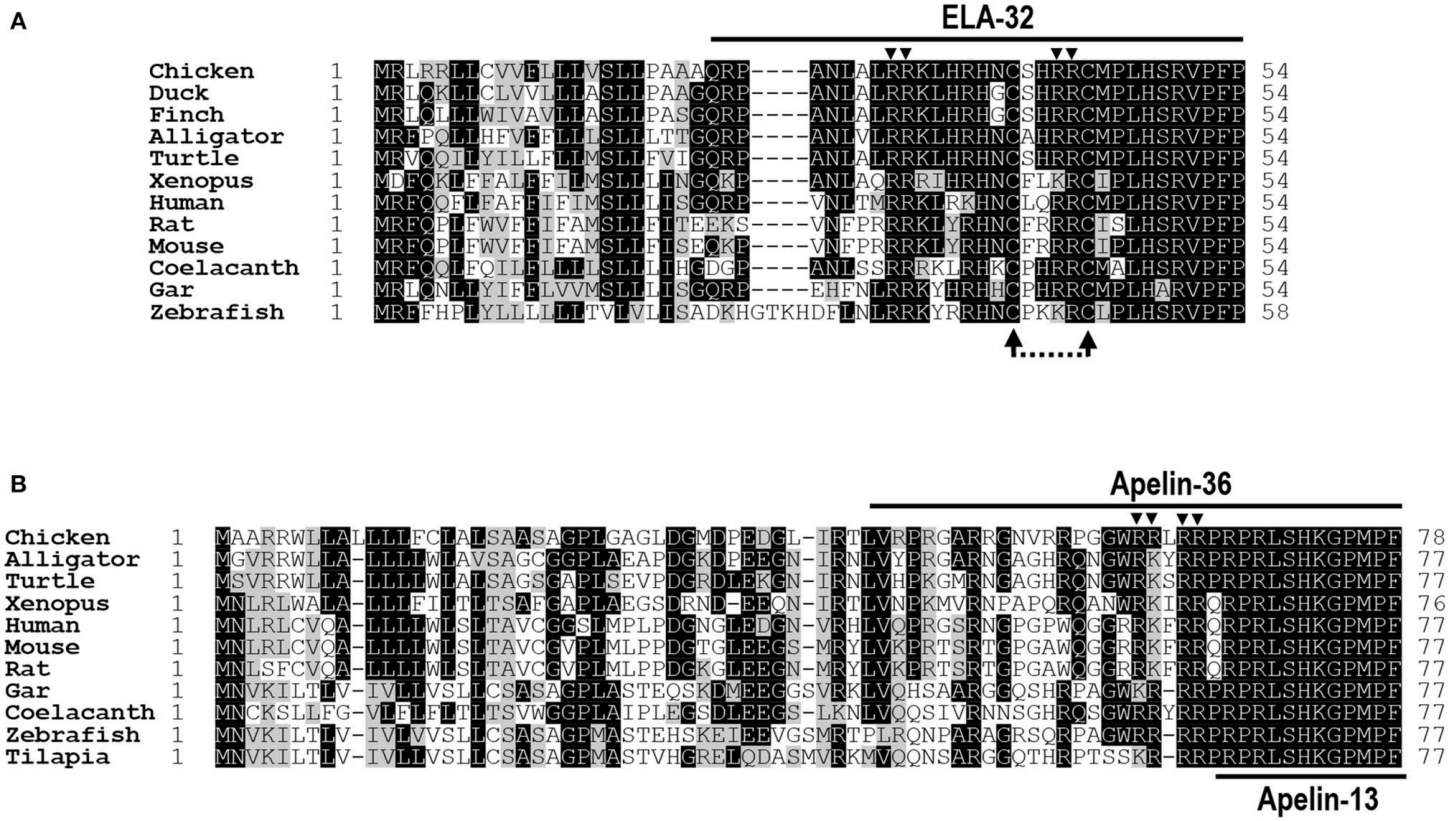
Alignment of ELA/apelin precursors of chickens and other species. **(A)** Amino acid alignment of chicken Elabela (ELA) precursor (KX017223) with that of ducks (XP_012952131), zebra finches (XP_012428876), American alligators (XP_014449490), Chinese soft-shelled turtles (XP_014430475), *Xenopus tropicalis* (XP_012811093), humans (NP_001284479), mice (NP_001284483), rats (XP_008770257), coelacanths (XP_014352760), spotted gars (XP_015199913), and zebrafish (NP_001284476). Arrowheads indicate the dibasic residues for proteolytic processing (i.e., R^31^R^32^; R^42^R^43^). The dotted arrows indicate the two cysteine residues, which may be required for a tentative intra-molecular disulfide bond formation in ELA peptides. **(B)** Amino acid alignment of chicken apelin precursor (KX017222) with that of American alligators (XP_014465437), western painted turtles (XP_005310961), *Xenopus tropicalis* (NP_001165146), humans (NP_059109), mice (NP_038940), rats (NP_113800), spotted gars (XP_015207270), coelacanths (XP_014341585), zebrafish (NP_001159596), and Nile tilapia (XP_013126785). Arrowheads denote the dibasic residues for proteolytic processing (i.e., R^61^R^62^, R^64^R^65^). All sequences were retrieved from the GenBank.

Although *APLN* and *ELA* were predicted to exist in birds, their full-length cDNAs have not been determined in any avian species. In this study, we cloned the full-length cDNAs of *APLN* and *ELA* from the chicken heart. Chicken *APLN* cDNA is 878 bp in length (KX017222) and predicted to encode a 78-amino acid (a.a.) precursor, which shows 42-73% a.a. sequence identities with preproapelin of other vertebrate species, including humans, turtles, *X. tropicalis*, spotted gars, and zebrafish. As in mammals, chicken apelin precursor is likely to produce multiple forms of mature apelin peptide, such as apelin-36 (36 amino acids) and apelin-13 (13 amino acids), after removal of its signal peptides and proteolytic processing at the dibasic residues (Figure 1; Supplementary Figure 1).

The cloned chicken *ELA* cDNA (KX017223) encodes a precursor of 54 amino acids, which shows 52–87% a.a. identities with that of ducks, zebra finches, American alligators, Chinese soft-shelled turtles, *X. tropicalis*, spotted gars, coelacanths, zebrafish, mice, rats, and humans. Like chicken apelin, cELA precursor also contains a signal peptide at its N-terminus, thus it is predicted to generate a 32-a.a. ELA peptide (ELA-32) after removal of its signal peptide. The mature chicken ELA-32 shows 63–97% a.a. sequence identities with ELA from other vertebrate species examined (Figure 1).

### Identification of APLNR1 and APLNR2 in Chickens and Red-Eared Sliders

Using human *APLNR* cDNA (NM_005161, also called *APLNR1* in this study) as the query sequence, we searched the genome database of chickens and western painted turtles. Interestingly, we identified two *APLNR*-like genes in chickens and turtles. Based on these sequence information, we designed primers to amplify and clone the cDNAs of the two *APLNRs* from chickens and red-eared sliders, confirming the existence of both genes in these species. According to their evolutionary origin, we named them as *APLNR1* and *APLNR2*, respectively.

**Figure 2:**
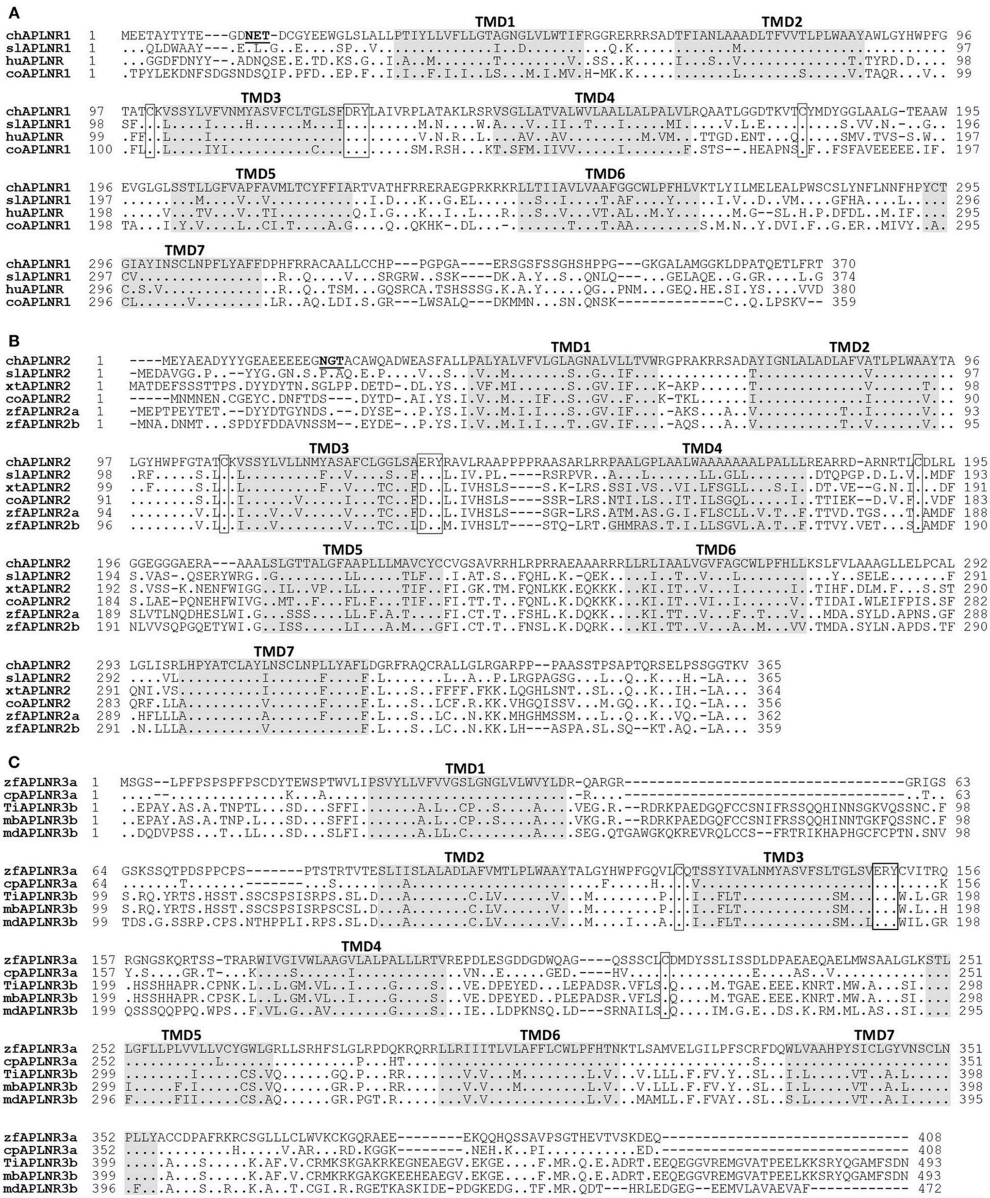
Alignment of APLNR(s) from chickens and other species. **(A)** Alignment of chicken APLNR1 (cAPLNR1, KU887746) with that of red-eared sliders (slAPLNR1, KU887748), humans (huAPLNR, NP_005152), and coelacanths (coAPLNR1, XP_005999317). **(B)** Alignment of chicken APLNR2 (cAPLNR2, KU887747) with that of red-eared sliders (slAPLNR2, KU887749), *Xenopus tropicalis* (xtAPLNR2, NP_001027492), coelacanths (coAPLNR2, XP_005990330), and zebrafish (zfAPLNR2a: NP_001068573; zfAPLNR2b; NP_001025368); **(C)** Alignment of zebrafish APLNR3a (zfAPLNR3a, KU887750) with that of common carp (cpAPLNR3a, KTG31429), or with APLNR3b of Nile tilapia (tiAPLNR3b, KU887751), zebra mbuna (mbAPLNR3b, XP_004563916), and medaka (mdAPLNR3b, XP_011474208). The conserved “D/ERY motif” and cysteine residues (for disulfide bond formation) are boxed and the seven transmembrane domains (TMD1-7) are shaded; the potential *N*-glycosylation site (NXT/S, X represents any residue except proline) is underlined; dots indicate amino acids identical to chicken APLNR1/APLNR2 or zebrafish APLNR3a; dashes denote gaps in the alignment.

Chicken *APLNR1* cDNA is predicted to encode a 370-a.a. receptor (KU887746), which shares high a.a. sequence identities with human APLNR (64%). Chicken *APLNR2* cDNA encodes a receptor of 365 a.a. (KU887747), which shows low amino acid identity (43%) with human APLNR. However, like human APLNR, both cAPLNR1 and cAPLNR2 contain seven transmembrane domains, a DRY/ERY motif near the end of the 3rd transmembrane domain, and a cysteine pair for disulfide bond formation known to be critical for receptor conformation (Figure 2).

The cloned red-eared slider APLNR1 (374 a.a., KU887748) and APLNR2 (365 a.a., KU887749) share high a.a. sequence identities with chicken APLNR1 (73%) and APLNR2 (67%), respectively. In addition, like chicken APLNR1 and APLNR2, they also share relatively high structural similarity with human APLNR (67; 47% identity) and contain many conserved structural motifs characteristic of class A GPCR, such as 7 transmembrane domains and an ERY motif (Figure 2; Supplementary Figure 2).

### Identification of a Novel APLNR (APLNR3) in Zebrafish, Tilapia, and Other Teleost Fish

**Figure 3:**
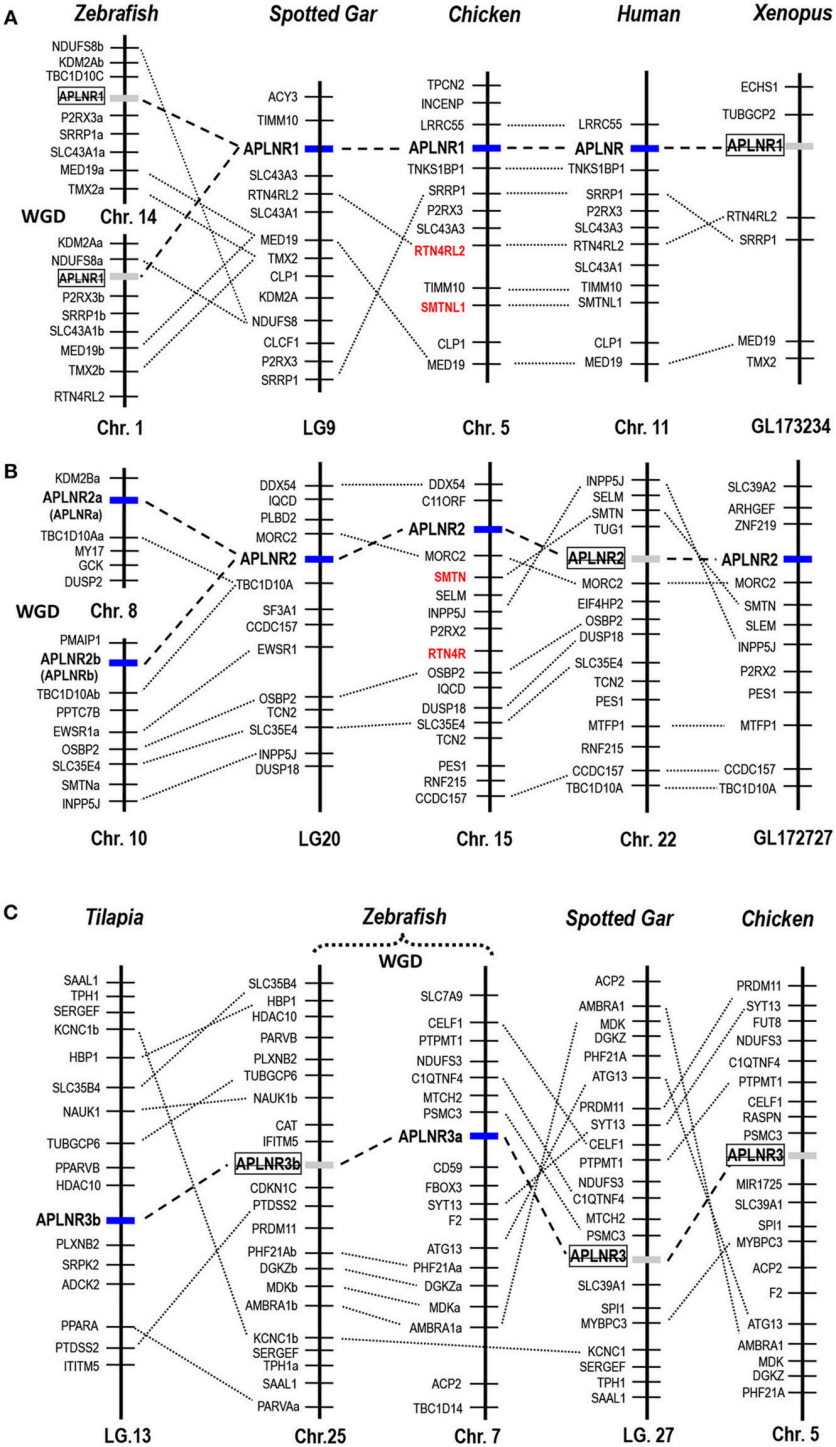
Synteny analysis of *APLNRs* in vertebrates. Synteny analyses show that **(A)** *APLNR1*, **(B)** *APLNR2*, and **(C)** *APLNR3 (APLNR3a or APLNR3b)* are located in three syntenic regions conserved in most vertebrate species examined. Dotted lines indicate the syntenic genes; dashed lines denote genes of interest. *APLNR1* exists in the genomes of humans, chickens and spotted gars, while it is likely lost in zebrafish and *Xenopus tropicalis*; *APLNR2* exists in zebrafish (*APLNR2a* and *APLNR2b*), spotted gars, *Xenopus tropicalis*, chickens, and opossums (Supplementary Figure 4), while it is likely lost in most mammalian species (including humans); *APLNR3* (*APLNR3a* or *APLNR3b*) exist in teleosts, but it is likely lost in tetrapods (e.g., chickens) and spotted gars. The existence of two copies of *APLNR2* (*APLNR2a, APLNR2b*) and *APLNR3* (*APLNR3a, APLNR3b*) in teleosts was likely originated from a teleost-specific genome duplication event (WGD) (53). Genes (colored in red) adjacent to *APLNR1/APLNR2* are paralogous (e.g., *SMTN-SMTNL, RTN4R-RTN4R2L*), which were likely generated by a whole genome duplication event during vertebrate evolution (54).

In this study, we also searched the genome database of zebrafish and identified three *APLNR*-like genes. Among the three *APLNR*-like genes, two of them have been reported previously. They are named *APLNRa* (aliased as *agtrl1a*, NP_001068573) and *APLNRb* (aliased as *agtrl1b*, NP_001025368), respectively (34, 35, 52). As revealed by synteny analysis shown in Figure 3, zebrafish *APLNRa* and *APLNRb* are orthologous to chicken *APLNR2*, but not to chicken *APLNR1* or human *APLNR* (*hAPLNR1*). Thus, we re-named zebrafish *APLNRa* and *APLNRb* as *APLNR2a* and *APLNR2b* respectively in this study. Interestingly, the third receptor gene we found encodes a novel APLNR-like receptor of 408 a.a. (KU887750), which shows low a.a. identities with human APLNR (36%), and zebrafish *APLNR2a*/*APLNR2b* (41%). Hence, this novel receptor was designated as *APLNR3a*.

As in zebrafish, both *APLNR2a* (*APLNRa*, XP_005473185) and *APLNR2b* (*APLNRb*, XP_013126265) were identified in Nile tilapia. In addition, we also identified and cloned a novel *APLNR*-like gene in tilapia. This gene encodes a receptor of 493 a.a. (KU887751), which shows relatively high a.a. sequence identity (57%) to zebrafish *APLNR3a*, but a low degree of identity to human APLNR (34%), zebrafish APLNR2s (~38%), and chicken APLNR1/2 (35–37%) (Supplementary Table 1). Thus, we named this receptor *APLNR3b*. Similar to the finding in zebrafish and tilapia, *APLNR3a* or *APLNR3b* could also be identified in a number of teleost fish, including medaka and fugu (Supplementary Figure 3).

Like human APLNR, zebrafish APLNR3a and tilapia APLNR3b contain 7 typical transmembrane domains, an ERY motif, and two cysteine residues for disulfide bond formation (Figure 2), indicating that APLNR3a/APLNR3b also belong to class A GPCR.

### Synteny Analysis of *APLNR* Gene(s) in Vertebrates

Using synteny analysis, we traced the evolutionary history of these APLNRs in vertebrate species including humans, chickens, *X. tropicalis*, zebrafish, and spotted gars. We found that chicken *APLNR1* is orthologous to human *APLNR*. This APLNR1 could also be identified in turtles, coelacanths and spotted gars, but it is likely lost in teleosts (e.g., zebrafish) and *X. tropicalis* (Figure 3).

Interestingly, *APLNR2* identified in chickens appears to be lost in humans and mice, but it can still be identified in all non-mammalian vertebrate species examined, including *X. tropicalis*, zebrafish and spotted gars. Furthermore, our synteny analysis indicates that zebrafish *APLNRa* and *APLNRb* reported in previous studies (34, 36–38, 52) are orthologous to chicken *APLNR2*. Hence, we re-name them as *APLNR2a* and *APLNR2b* respectively (Figure 3). Moreover, we also found an *ALPNR2* fragment in the opossum genome (Supplementary Figure 4), indicating the existence of *APLNR2* in some mammalian species.

Although *APLNR3a/APLNR3b* could be identified in teleosts, it is likely lost in tetrapods including chickens, as evidenced by synteny analysis.

### Functional Characterization of APLNRs From Chickens, Turtles, and Zebrafish

**Figure 4:**
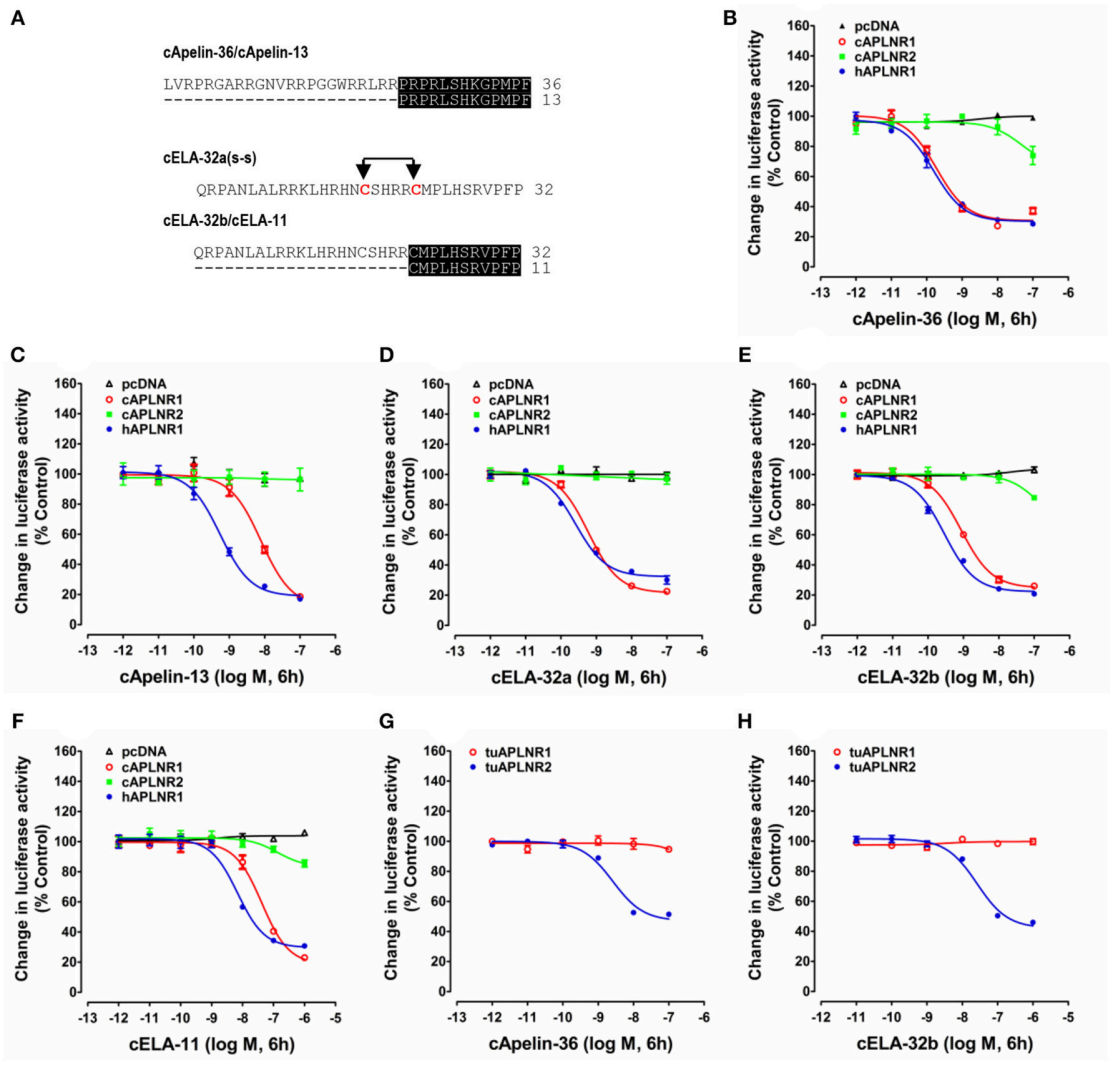
Functional analysis of chicken, human and turtle APLNR(s). **(A)** Amino acid sequences of chicken apelin-36 (36 amino acids), apelin-13 (13 amino acids), ELA-32a (32 amino acids with the tentative disulfide bond), ELA-32b (without the tentative disulfide bond), and ELA-11 peptides used in this experiment. The identical C-terminal regions between apelin-36 and apelin-13, or between ELA-32b and ELA-11, are shaded. **(B–F)** Effects of chicken apelin-36 **(B)**, apelin-13 **(C)**, ELA-32a **(D)**, ELA-32b **(E)**, and ELA-11 **(F)** (10^−12^ to 10^−7^ M, or 10^−12^ to 10^−6^ M, 6 h) on forskolin (5 μM)-stimulated luciferase activity of HEK293 cells expressing chicken APLNR1 (cAPLNR1), APLNR2 (cAPLNR2), or human APLNR (hAPLNR1), monitored by a pGL3-CRE-luciferase reporter system. **(G,H)** Effects of chicken apelin-36 **(A)** and ELA-32b **(B)** on forskolin (5 μM)-stimulated luciferase activity of HEK293 cells expressing red-ear slider APLNR1 (tuAPLNR1) and APLNR2 (tuAPLNR2), monitored by a pGL3-CRE-luciferase reporter system. As a negative control, peptide treatment did not inhibit forskolin-stimulated luciferase activity of HEK293 cells transfected with the empty pcDNA3.1(+) vector. Each data point represents mean ± SEM of three replicates (*N* = 3).

To test whether APLNRs identified in chickens, turtles and zebrafish are functional, each receptor was transiently expressed in HEK293 cells and treated with various concentrations of synthetic chicken peptides [apelin-36, apelin-13, ELA-32a (with the intra-molecular disulfide bond), ELA-32b (without the disulfide bond) and ELA-11] (Figure 4A). The receptor activation (or inhibition) of the cAMP and MAPK signaling pathways were then monitored by pGL3-CRE-luciferase or pGL4-SRE-luciferase reporter systems, respectively, as established in our previous studies (48–51).

As shown in Figure 4, using pGL3-CRE-luciferase reporter system, we demonstrated that chicken apelin-36, apelin-13, ELA-32a, ELA-32b, and ELA-11 treatment dose-dependently inhibited forskolin (5 μM)-stimulated luciferase activities of HEK293 cells via activating chicken APLNR1. The order of the potencies of these peptides in activating cAPLNR1 is: apelin-36 (EC_50_: 0.18 ± 0.10 nM) > ELA32a (EC_50_: 0.59 ± 0.13 nM) ≈ ELA32b (EC_50_: 0.87 ± 0.23 nM) > apelin-13 (EC_50_: 7.86 ± 2.73 nM) > ELA-11 (EC_50_: 42.1 ± 18.96 nM). As a positive control, all peptides could dose-dependently inhibit forskolin-induced luciferase activities of HEK293 cells expressing human APLNR (Table 1). These findings clearly indicate that like human APLNR, chicken APLNR1 can function as a receptor common for apelin and ELA peptides and is functionally coupled to Gi-cAMP signaling pathway. Unlike cAPLNR1, cAPLNR2 could only be activated by high concentrations of apelin-36/ELA peptides (≥100 nM), implying that cAPLNR2 could not transmit signals effectively *in vitro*.

**Table 1:** EC_50_ values of chicken (c) and zebrafish (zf) apelin/ELA peptides in activating APLNR(s) of chickens (c), humans (h), zebrafish (zf), tilapia (ti), and red-eared sliders (tu), examined by the pGL3-CRE-luciferease reporter assay.

**Ligands**	**EC**_****50****_ **(nM)**								
	**hAPLNR**	**cAPLNR1**	**cAPLNR2**	**zfAPLNR2a**	**zfAPLNR2b**	**zfAPLNR3a**	**tiAPLNR3b**	**tuAPLNR1**	**tuAPLNR2**
cApelin-36	0.16 ± 0.06	0.18 ± 0.10	>100	2.96 ± 1.26	0.90 ± 0.33	1.88 ± 0.54	1.36 ± 0.96	N	2.58 ± 1.36
cApelin-13	0.55 ± 0.21	7.86 ± 2.73	N	2.82 ± 1.18	12.7 ± 5.21	>100	174 ± 115.61	–	–
cELA-32a	0.26 ± 0.07	0.59 ± 0.13	N	1.81 ± 0.74	2.55 ± 0.11	N	N	–	–
cELA-32b	0.28 ± 0.05	0.87 ± 0.23	>100	1.68 ± 0.58	1.02 ± 0.23	N	N	N	26.4 ± 10.11
cELA-11	6.94 ± 2.93	42.1 ± 18.96	N	>100	>100	N	N	–	–
zfApelin-36	–	–	–	0.89 ± 0.44	0.51 ± 0.31	0.45 ± 0.12	0.77 ± 0.24	–	–
zfELA-22	–	–	–	1.43 ± 0.57	1.78 ± 1.17	>100	N	–	–
zfELA-36	–	–	–	1.61 ± 0.80	1.29 ± 0.90	>100	N	–	–

*“N” means that the EC_50_ (mean ± S.E. of 3 independent experiments) value cannot be evaluated based on the experimental data; cELA-32a denotes the synthetic chicken ELA-32 peptide with the tentative intra-molecular disulfide bond; cELA-32b represents the synthetic peptide without the tentative intra-molecular disulfide bridge; zfELA-36/zfELA-22 represents the synthetic peptides of 36/22 a.a. without the intra-molecular disulfide bridge (see Figure 1 for details); ‘–', not tested*.

Strikingly, in red-eared sliders, APLNR2 (but not APLNR1) expressed in HEK293 cells could be potently activated by apelin-36 and ELA-32 (Table 1), and its activation results in the inhibition of forskolin-stimulated luciferase activities (Figure 4), suggesting that APLNR2, rather than APLNR1, is likely the functional receptor common for both peptides in turtles.

**Figure 5:**
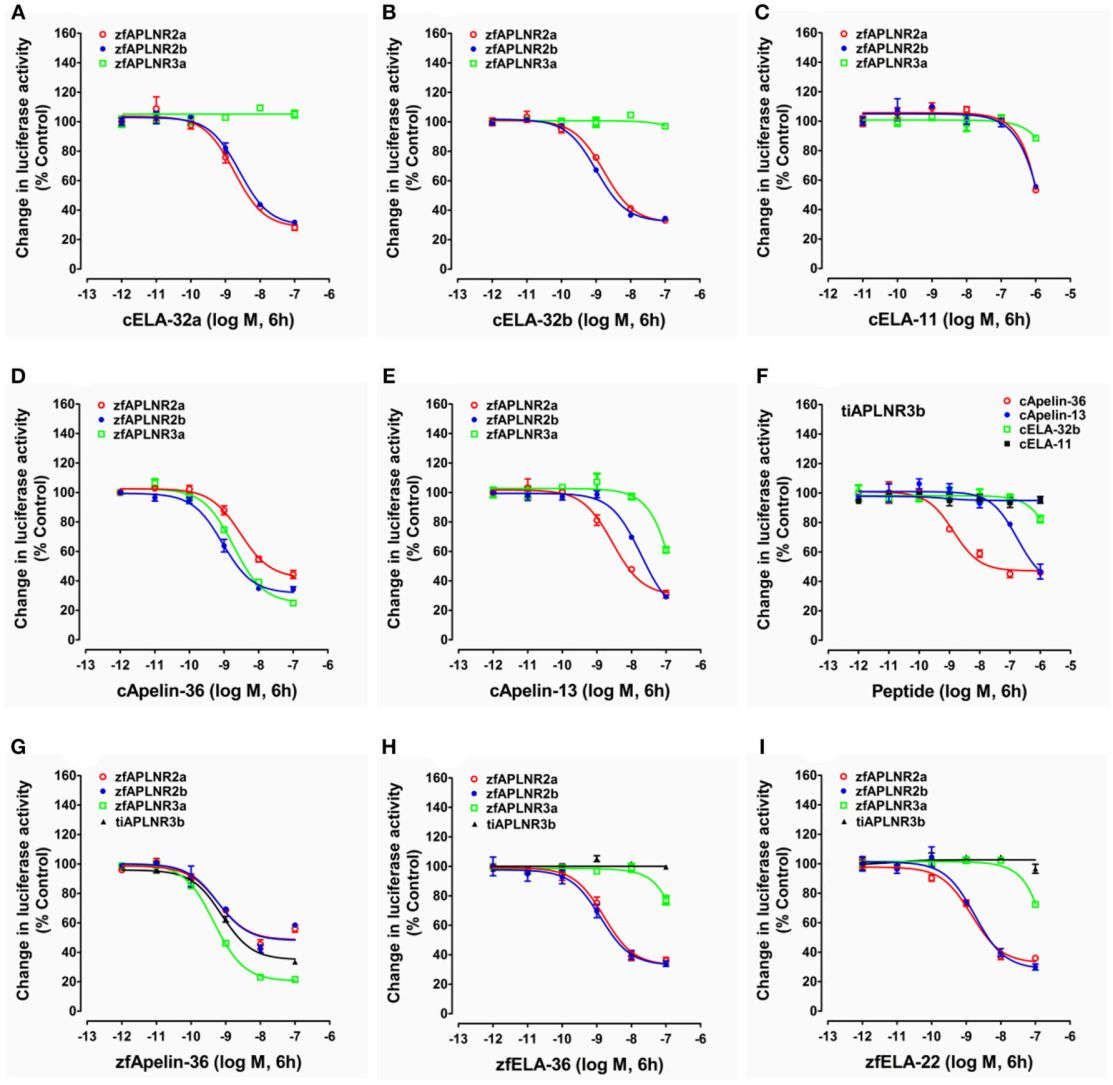
Functional analysis of zebrafish APLNRs. **(A–E)** Effects of chicken ELA-32a **(A)**, ELA-32b **(B)**, ELA-11 **(C)**, apelin-36 **(D)**, and apelin-13 **(E)** on forskolin (5 μM)-stimulated luciferase activity of HEK293 cells expressing zebrafish APLNR2a (zfAPLNR2a), APLNR2b (zfAPLNR2b), and APLNR3a (zfAPLNR3a), monitored by a pGL3-CRE-luciferase reporter system. **(F)** Effects of chicken apelin and ELA peptides (10^−12^ to 10^−6^ M, 6h) on forskolin (5 μM)-stimulated luciferase activity of HEK293 cells expressing tilapia APLNR3b (tiAPLNR3b), monitored by a pGL3-CRE-luciferase reporter system. **(G–I)** Effects of zebrafish apelin (**G**: zfApelin-36) and ELA (**H**: zfELA-36; **I**: zfELA-22) peptides on forskolin (5 μM)-stimulated luciferase activity of HEK293 cells expressing zebrafish (zf) APLNR2a/APLNR2b/APLNR3a or tilapia APLNR3b (tiAPLNR3b), monitored by a pGL3-CRE-luciferase reporter system. Each data point represents mean ± SEM of three replicates (*N* = 3).

In zebrafish, APLNR2a (APLNRa), and APLNR2b (APLNRb) could be activated by chicken apelin-36, apelin-13, ELA-32a, and ELA-32b with high potencies, whereas ELA-11 is much less potent (Figure 5; Table 1). These findings suggest that both receptors may function as the receptors common to apelin and ELA peptides. Interestingly, zebrafish APLNR3a could be potently activated by chicken apelin-36 (EC_50_: 1.88 ± 0.54 nM), but not by ELA peptide, suggesting that APLNR3a may function as a receptor specific to apelin. Like zebrafish APLNR3a, tilapia APLNR3b could also be potently activated by apelin-36 (EC_50_: 1.36 ± 0.96 nM), but not by ELA-32, suggesting that APLNR3b may also function as an apelin-specific receptor (Figure 5). In support of this notion, we further demonstrated that zebrafish APLNR3a or tilapia APLNR3b expressed in HEK293 cells could be potently activated by synthetic zebrafish apelin-36, but not by zebrafish ELA-36/ELA-22, whereas zebrafish APLNR2a/APLNR2b could be activated potently by both apelin and ELA (Figure 5; Table 1).

**Figure 6:**
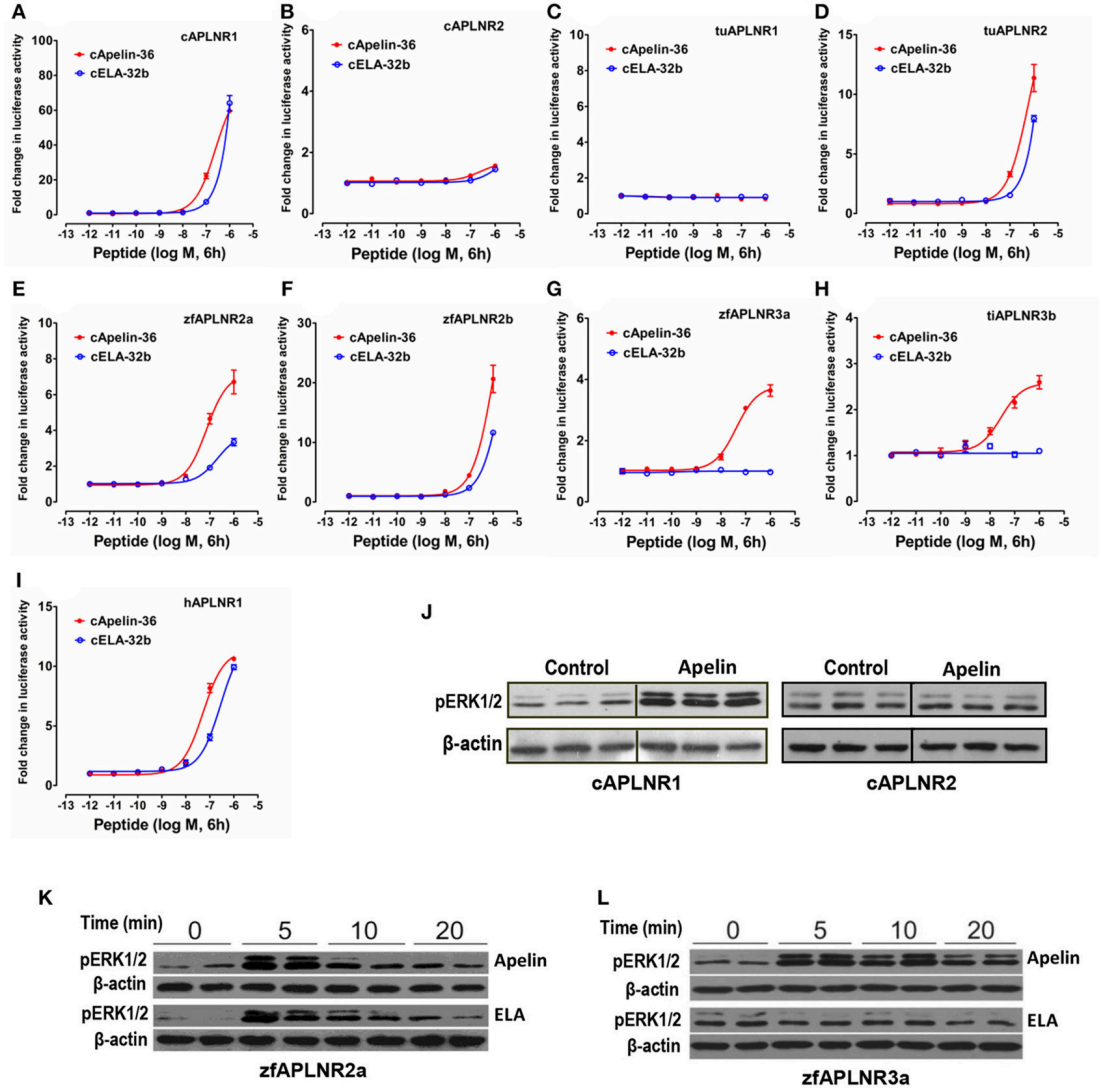
Functional coupling of vertebrate APLNRs to the MAPK signaling pathway. **(A–I)** Functional characterization of chicken APLNR1 (**A**: cAPLNR1) and APLNR2 (**B**: cAPLNR2), red-eared slider APLNR 1 (**C**: tuAPLNR1) and APLNR2 (**D**: tuAPLNR2), zebrafish APLNR2 (**E**: zfAPLNR2a; **F:** zfAPLNR2b) and APLNR3a (**G:** zfAPLNR3a), and tilapia APLNR3b (**H:** tiAPLNR3b), and human APLNR (**I:** hAPLNR1) using a pGL4-SRE luciferase reporter system. The receptor-activated MAPK signaling pathway was monitored by a system of co-transfection of pGL4-SRE luciferase reporter construct and the respective receptor expression plasmid in HEK293 cells upon 6-h treatment of apelin-36 or ELA-32b (10^−12^ to 10^−6^ M). Each data point represents mean ± SEM of three replicates (*N* = 3). **(J)** Western blot showed that in HEK293 cells expressing chicken APLNR1 (not cAPLNR2), chicken apelin-13 treatment (100 nM, 10 min, 3 replicates) can strongly enhance ERK1/2 phosphorylation (pERK1/2). **(K)** Both zebrafish apelin-36 and ELA-36 treatment (100 nM, duplicates) can enhance phosphorylation of ERK1/2 in HEK293 cells expressing zebrafish APLNR2a in a time-dependent manner (5, 10, 20 min). **(L)** In HEK293 cells expressing zebrafish APLNR3a, zebrafish apelin-36, not ELA-36, can induce ERK1/2 phosphorylation.

Using the pGL4-SRE-luciferase reporter system (Figure 6), we demonstrated that like human APLNR (hAPLNR1), chicken APLNR1, zebrafish APLNR2a, and APLNR2b, turtle APLNR2, and zebrafish APLNR3a, and tilapia APLNR3b are functionally coupled to MAPK/ERK signaling cascade. In agreement with this finding, Western blot analysis also revealed that apelin/ELA treatment could rapidly enhance the ERK1/2 phosphorylation in HEK293 cells expressing chicken APLNR1/zebrafish APLNR2a/APLNR3a (Figures 6J–L). In sharp contrast, chicken APLNR2 and turtle APLNR1 showed little or no response to peptide treatment (Figure 6).

### Tissue Expression of *APLNRs, APLN*, and *ELA* in Chickens and Zebrafish

**Figure 7:**
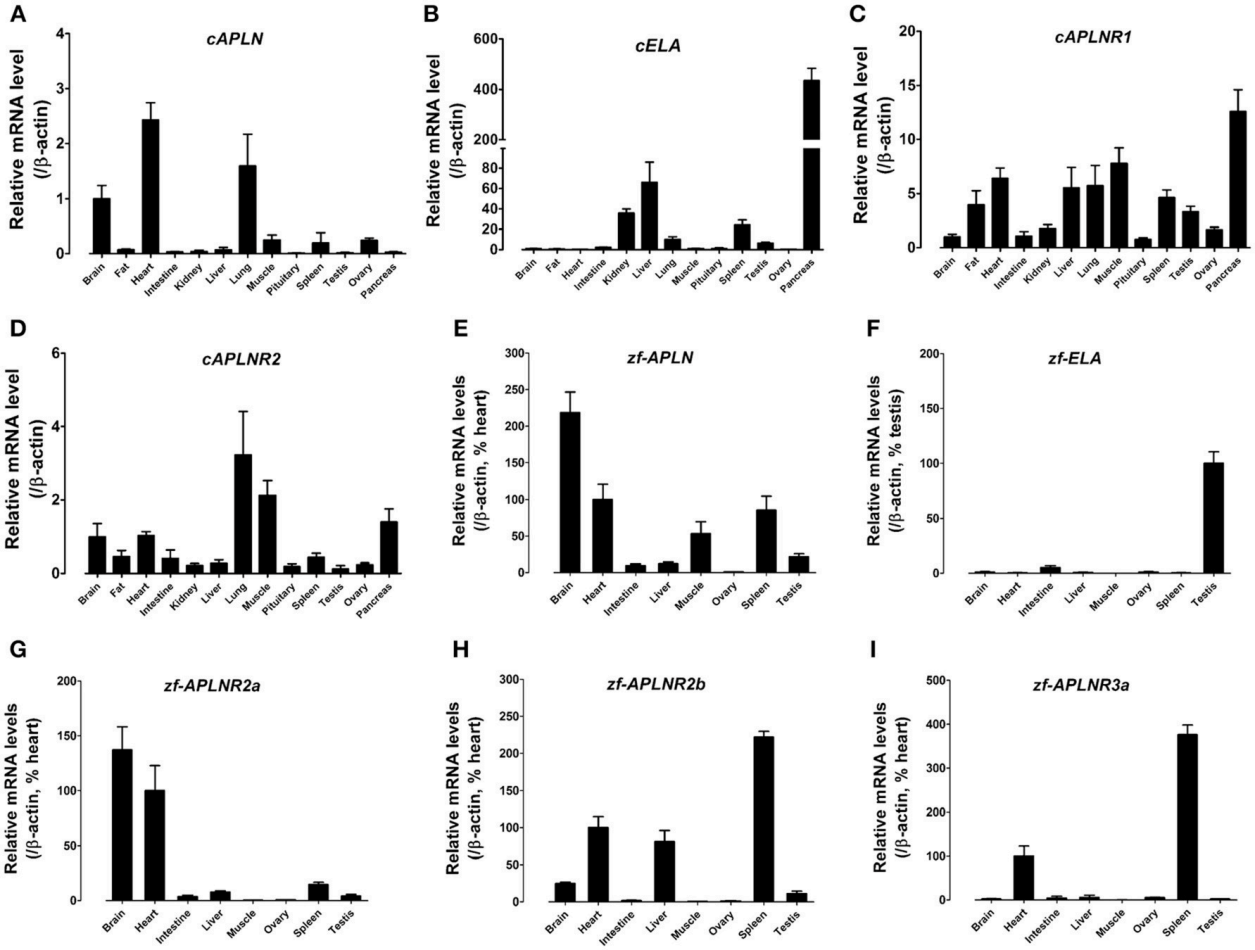
Tissue expression of *APLN, ELA*, and *APLNRs* in adult chickens and zebrafish. **(A–D)** Quantitative real-time RT-PCR assay of *APLN* **(A)**, *ELA* **(B)**, *APLNR1* **(C)**, and *APLNR2* **(D)** mRNA levels in adult chicken tissues. The mRNA level of target genes were normalized by β-actin and expressed as the relative fold difference compared to that of whole brain tissue. **(E–I)** Quantitative real-time RT-PCR assay of *APLN* **(E)**, *ELA* **(F)**, *APLNR2a* **(G)**, *APLNR2b* **(H)**, and *APLNR3a* **(I)** mRNA levels in adult zebrafish tissues. The mRNA level of target genes were normalized by that of β-actin and expressed as the percentage of zebrafish heart or testis. Each data point represents the mean ± SEM of four adult chickens/zebrafish (*N* = 4).

Using quantitative real-time PCR (qPCR), we examined the mRNA expression of *APLN, ELA, APLNR1*, and *APLNR2* (Figures 7A–D), in 13 chicken tissues. Both *cAPLNR1* and *cAPLNR2* were detected to be widely expressed in all chicken tissues examined, including the brain, abdominal fat, heart, lung, muscle, spleen and pancreas. Like *cAPLNRs, cAPLN*, and *cELA* were detected to be widely expressed in chicken tissues. *cAPLN* is abundantly expressed in the brain, heart and lung, and weakly expressed in other remaining tissues, whereas *cELA* is expressed highly in the pancreas and liver, moderately in the kidneys, lung, spleen and testes, and weakly in other tissues examined.

Zebrafish *APLN, ELA*, and *APLNRs* are reported to play crucial roles in cardiovascular development during embryogenesis (34–38), however, their expression has not been fully characterized in adult zebrafish tissues. Hence, using qPCR, we examined the mRNA expression of *APLN, ELA*, and *APLNRs* in adult zebrafish tissues (Figure 7). As shown in Figure 7, *APLN* is widely expressed in all tissues examined, with a relatively higher expression noted in the brain, heart, muscle and spleen. Interestingly, unlike *APLN, ELA* is expressed predominantly in the testes and weakly in other tissues examined including the intestine, brain and heart. Like *APLN* and *ELA*, the three *APLNRs* were found to be widely, but differentially, expressed in zebrafish tissues. *APLNR2a* (alias: *APLNRa*) is highly expressed in the brain and heart and weakly in other tissues examined. *APLNR2b* (alias: *APLNRb*) is highly expressed in the heart, liver and spleen, moderately in the brain and testes, and weakly in other tissues. *APLNR3a* is highly expressed in the spleen and heart, and weakly in other tissues examined (Figure 7).

**Figure 8:**
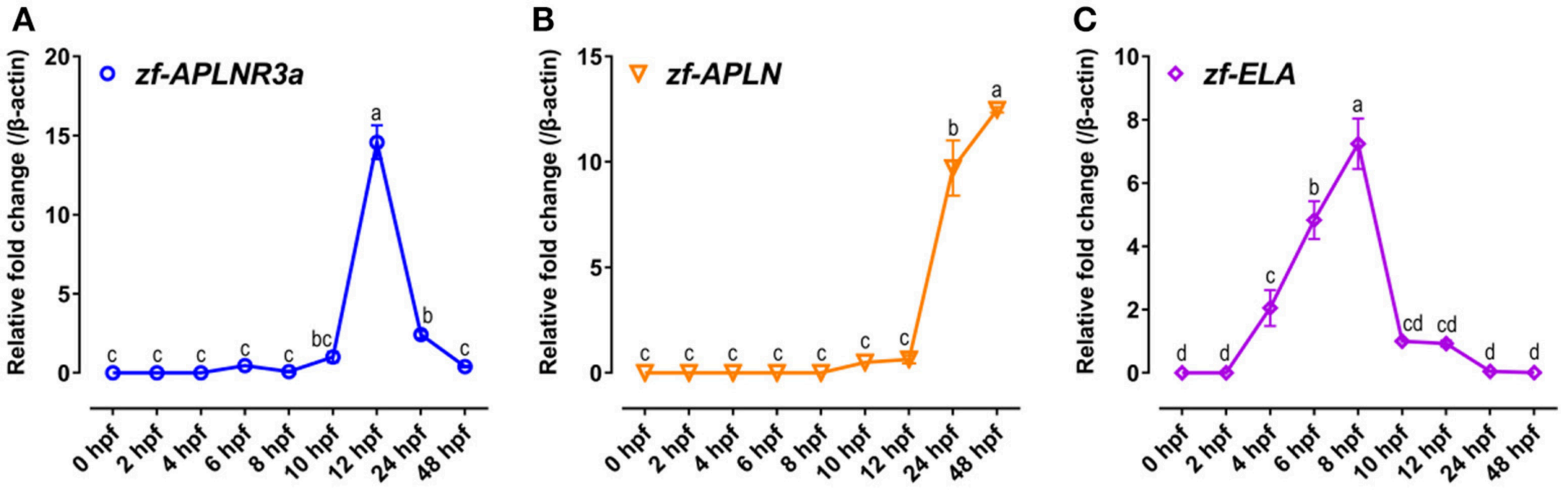
Expression of *APLNR3a, APLN*, and *ELA* in zebrafish embryos. **(A–C)** qPCR assay of *APLNR3a* **(A)***, APLN* **(B)**, and *ELA* **(C)** mRNA expression in zebrafish embryos at different stages (0, 2, 4, 6, 8, 10, 12, 24, and 48 hpf). The mRNA levels of target genes were normalized by β-actin and expressed as the fold change compared with that of 10 hpf embryos. hpf, hour post-fertilization. Each data point represents as mean ± SEM of 6–15 embryos (*N* = 6–15). Values significantly different (*P* < 0.05) between stages are indicated by different letters.

Since APLNR3a is a novel apelin-specific receptor, thus, the mRNA expression of *APLNR3a* in developing zebrafish embryos was also examined to determine whether like APLNR2s, APLNR3a signaling is involved in zebrafish embryogenesis. As shown in Figure 8, the mRNA level of *APLNR3a* is low at early embryonic stages (0–10 hpf), but increases rapidly at 12 hpf stage (~15-fold increase vs. 10 hpf), and then drops sharply at later stages (24 and 48 hpf).

## Discussion

In this study, we identified and characterized two APLNRs in chickens and turtles. Moreover, we identified three functional APLNRs in zebrafish, including a novel apelin-specific receptor. To our knowledge, this study represents the first to systematically characterize all APLNRs in several representative vertebrate species, including chickens and zebrafish. Undoubtedly, our data will aid to elucidate the physiological roles of APLNRs and their ligands in different classes of vertebrates.

### Identification of *APLN, ELA, APLNR1*, and a Novel *APLNR2* in Chickens

In this study, we cloned the full-length cDNAs of *APLN, ELA, APLNR1*, and *APLNR2* from chicken heart. Like human *APLN*, chicken *APLN* cDNA consists of three exons, including a non-coding exon at its 3′-UTR (Supplementary Figure 1A) (3). It encodes a preproapelin of 78 a.a. As in mammals, chicken preproapelin may generate multiple forms of apelin, such as apelin-36, apelin-16, and apelin-13, after proteolytic processing at various dibasic residues (e.g., R^61^R^62^, R^64^R^65^) (Figure 1B). It is reported that pyroGlu-apelin-13 is predominantly synthesized in rat brain or human cardiac tissue (19, 55). This molecule displays strong biological activity both *in vitro* and *in vivo*, however the first residue at the N-terminus of apelin-13 is Pro in chickens and other non-mammalian species, instead of Gln, indicating that pyroGlu-apelin-13 does not exist in these species (Figure 1B).

Like *cAPLN*, chicken *ELA* cDNA contains three exons and encodes a precursor of 54 a.a. (Supplementary Figure 1B). Similar to zebrafish ELA, chicken ELA precursor may generate multiple forms of ELA peptides, such as ELA-32, ELA-22, and ELA-11 after removal of its signal peptide and proteolytic processing at multiple dibasic residues (R^31^R^32^; R^42^R^43^) (Figure 1) (37). Two cysteine residues were found to be fully conserved among ELA-32 of all vertebrate species examined. This led us to speculate that an intra-molecular disulfide bond (Cys^39^-Cys^44^) may exist in ELA-32 peptide and it may be required for its full biological activity. However, our functional study prove that the absence of this disulfide bond does not impair the biological activity of ELA-32, in view of the comparably high potencies of both forms of ELA-32 in activating human APLNR, chicken APLNR1 and zebrafish APLNR2s (Figures 4, 5).

In this study, we identified two APLNRs in chickens. cAPLNR1 is orthologous to human APLNR and shows structural conservation with its mammalian counterparts, including a DRY motif characteristic of class A GPCR family and a serine/threonine-rich C-terminus critical for receptor internalization and desensitization (56, 57). Apart from cAPLNR1, a novel APLNR-like receptor (named APLNR2) was also identified in chickens. It shows 41–48% a.a. sequence identity with cAPLNR1 and human APLNR. Like chicken APLNR1, chicken APLNR2 contains conserved structural motifs, such a D/ERY motif and a serine/threonine-rich C-terminal tail (56) (Figure 2).

### Identification of *APLNRs* in Other Non-mammalian Vertebrates: Implications for Their Evolutionary History

**Figure 9:**
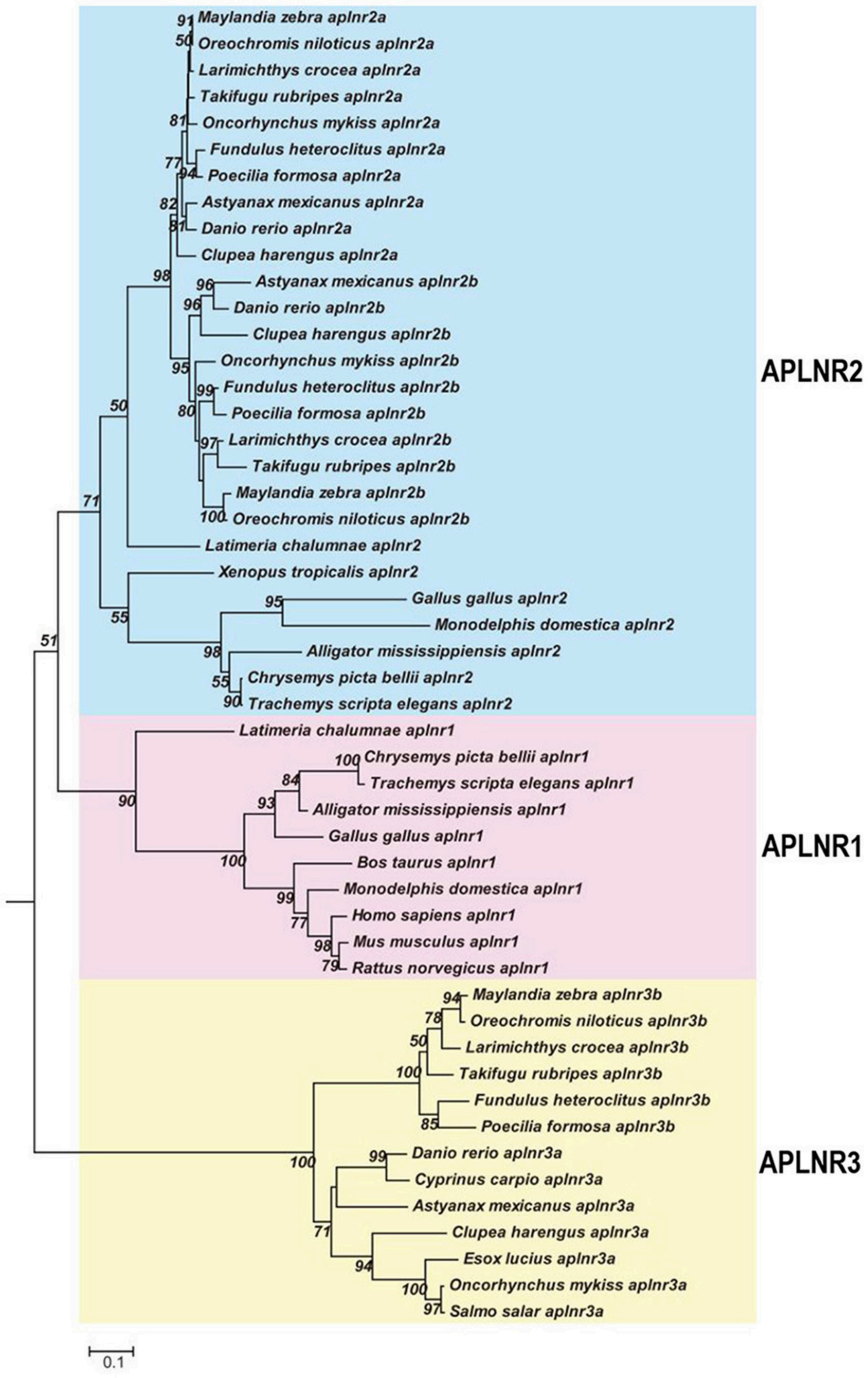
Phylogenetic analysis of vertebrate APLNRs. Phylogenetic tree (constructed by Neighboring-Joining method) shows the evolutionary relationship of APLNR1, APLNR2 (APLNR2a/APLNR2b), and APLNR3 (APLNR3a/APLNR3b) from chickens and other vertebrate species. Numbers near each branch point indicates the bootstrap values. The amino acid sequences of *APLNRs* from different species were retrieved from the GenBank.

**Figure 10:**
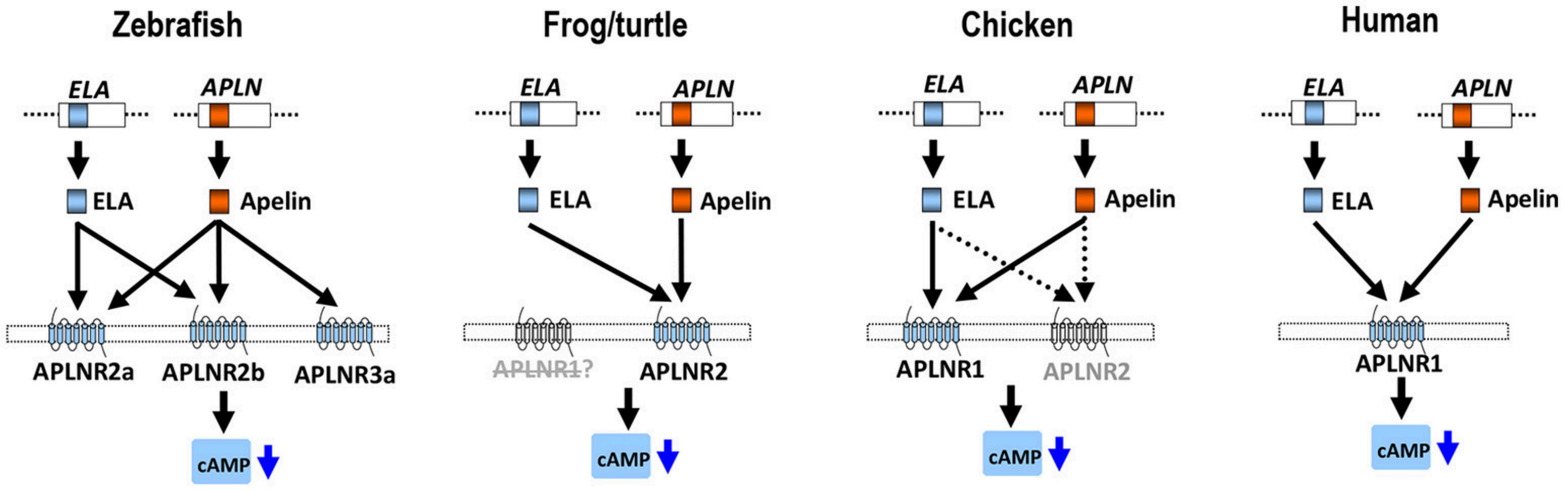
APLNR(s) in representative vertebrate species. In zebrafish, three functional APLNRs (APLNR2a, APLNR2b, and APLNR3a) for apelin/ELA peptides (encoded by *APLN*/*ELA* genes) were identified. Among them, APLNR2a (APLNRa) and APLNR2b (APLNRb) are receptors common for ELA and apelin, while APLNR3a is an apelin-specific receptor. In *Xenopus tropicalis* (or turtles), APLNR2 may act as a receptor common for both peptides, while APLNR1 may be either lost, or non-functional. In chickens, APLNR1 and APLNR2 have been identified. APLNR1 can be activated by apelin and ELA potently and thus functions as a receptor common to both peptides, whereas APLNR2 can only be activated by both peptides at high concentrations, implying it has a less significant role in mediating apelin/ELA actions. In humans, only a single APLNR (APLNR1) exists and it can be activated by apelin/ELA potently, indicating a crucial role of APLNR1 in mediating apelin/ELA actions. Activation of these APLNR(s) can activate multiple intracellular signaling pathways, including the inhibition of cAMP signaling pathway and activation of the MAPK/ERK signaling cascade (2).

As in chickens, two (or three) *APLNR*s homologous to human *APLNR* were identified in other non-mammalian vertebrates including turtles, coelacanths, spotted gars, zebrafish and tilapia, and we designated them as *APLNR1, APLNR2*, and *APLNR3*, respectively. Synteny analyses further revealed that *APLNR1, APLNR2*, and *APLNR3* are localized in three separate syntenic regions conserved in vertebrates (Figure 3). This finding led us to hypothesize that an ancestral vertebrate *APLNR* may have undergone gene/genome duplication events, resulting in the emergence of multiple *APLNRs* in modern vertebrates (54). The relatively high structural and functional similarity between vertebrate APLNR1 and APLNR2 also hints that APLNR1 and APLNR2 were most likely originated from a recent genome duplication event. This idea is supported by the identification of many paralogous genes adjacent to *APLNR1*/*APLNR2*, such as *SMTN*/*SMTNL1* and *RTN4R*/*RTN4RL2*. Moreover, the hypothetic teleost-specific genome duplication event (3R) which occurred around 350 million years ago (mya) (53) may have given rise to the two copies of *APLNR2* (*APLNR2a* and *APLNR2b*) and *APLNR3* (*APLNR3a*/*APLNR3b*) in teleosts. Our hypotheses are further supported by the phylogenetic analysis, in which *APLNRs* from different vertebrate species were clustered into three separate clades, namely the APLNR1 clade, APLNR2 clade (APLNR2a/APLNR2b), APLNR3 clade (APLNR3a/APLNR3b), and APLNR1 and APLNR2 clades show a much closer evolutionary relationship to each other (Figure 9). Although multiple *APLNRs* are expected to exist in modern vertebrates, lineage-specific gene loss event(s) may have caused the varying numbers of *APLNRs* in different vertebrate species (Figures 3, 10). Clearly, the identification of *APLNRs* from additional vertebrate species in the future will help to uncover the evolutionary history of *APLNRs* in finer details.

### APLNR1 Is a Functional Receptor Common for Apelin and ELA in Chickens and Humans

In this study, we found that chicken APLNR1 can be potently activated by apelin-36 and ELA-32 with high potencies and its activation results in the inhibition of the cAMP signaling pathway, indicating that like human APLNR (40, 41), cAPLNR1 is a receptor common for both peptides and coupled to the Gi-cAMP signaling pathway.

It is reported that in mammals, apelin-13 is a potent ligand for APLNR (3) and regulates cardiovascular function. In this study, we demonstrated that chicken apelin-13 and apelin-36 have similarly high potencies in activating human APLNR, however, chicken apelin-13 is 40-fold less potent than apelin-36 in activating chicken APLNR1. This interesting finding implies that unlike that in mammals, the long form(s) of apelin (e.g., apelin-36), rather than apelin-13, may be the potent ligand of chicken APLNR1 (3, 19). Similarly, we also noted that the long form of ELA (e.g., ELA-32) is much more potent than the short form (i.e., ELA-11), hinting that the long form(s) of ELA (e.g., ELA-32/ELA-22) may be the potent ligand(s) of cAPLNR1. Clearly, the isolation of apelin/ELA peptides from chicken tissues in the future will help to define the precise structure of endogenous ligands of cAPLNR1.

Although chicken APLNR2 shows 48% a.a. sequence identity to chicken APLNR1, it could only be activated by chicken apelin-36 or ELA-32 at high concentrations (≥100 nM) far beyond the physiological range. This finding implies that APLNR2 may play a less significant role in mediating apelin/ELA actions in chickens *in vivo*.

### APLNR2 Is a Functional Receptor Common for Apelin and ELA in Turtles/Zebrafish: A Functional Switch Between APLNR1 and APLNR2 During Vertebrate Evolution

The ineffectiveness of chicken APLNR2 in signal transmission led us to further question whether APLNR2 of turtles or zebrafish are functional. Strikingly, we found that turtle APLNR2, but not APLNR1, can be activated by apelin-36 and ELA-32 potently. This suggests that in turtles, APLNR2 may act as a common receptor for both peptides. Similarly, both zebrafish APLNR2a and APLNR2b are potently activated by apelin and ELA (Table 1), indicating that both receptors can act as receptors common to both peptides, and are functionally coupled to the Gi-cAMP signaling pathway, as previously proposed (37, 38). Interestingly, we also noted that only *APLNR2* exists in *X. tropicalis* genome, while *APLNR1* is likely lost, as evidenced by our synteny analysis (Figure 3). Moreover, this APLNR2 has been proven to be a functional receptor in *Xenopus laevis* and bull frogs, which can be activated by apelin-13 *in vitro* (33, 58). In view of the facts that: (1) APLNR2 is functional in zebrafish, frogs and turtles, and APLNR1 is likely lost, or non-functional in these species; (2) APLNR1 is functional in chickens and humans, we hypothesize that there exists a dramatic functional switch between APLNR1 and APLNR2 in mediating apelin/ELA actions across vertebrates. In zebrafish and frogs, APLNR2 plays a key role in mediating apelin/ELA actions, while in birds and mammals, APLNR1 (aliased as APLNR in humans) may play a crucial role in mediating actions of both peptides (Figure 10). The functional switch between APLNR1 and APLNR2 reminds us that the reported actions of APLNR(s) in teleosts, frogs and mammals should be interpreted with caution, since *APLNR1* and *APLNR2, per se*, are non-identical genes, both of which were likely duplicated from a common ancestral *APLNR* gene around 400 mya and have diverged for their own specific physiological functions ever since (54).

### Existence of a Novel Functional Apelin-Specific Receptor in Zebrafish and Tilapia

In this study, a novel APLNR, named APLNR3a, was identified in zebrafish. Functional assay proved that APLNR3a is an apelin-specific receptor and functionally coupled to both Gi-cAMP and MAPK signaling pathways. Similarly, we also identified a novel functional receptor (APLNR3b) specific to apelin in tilapia. To our knowledge, this study represents the first to report an apelin-specific receptor in vertebrates. The high structural and functional similarity of APLNR3a and APLNR3b also supports our hypothesis that *APLNR3a* and *APLNR3b* were likely generated by a teleost-specific genome duplication event (Figure 3) (53).

### Expression of *ALPN/ELA/APLNRs* in Chickens and Zebrafish

As a functional receptor common for apelin and ELA, *cAPLNR1* is widely expressed in all chicken tissues examined (Figure 7). This finding is consistent with the wide distribution of *APLNR* in humans, rats and mice (4, 7). It also suggests that APLNR1 can mediate the actions of apelin/ELA in various chicken tissues. As in rats and mice (6, 7, 28, 40), *cAPLN* mRNA is also widely expressed in all tissues examined with a high mRNA level noted in the brain, heart and lung. Similarly, *APLN* has also been reported to be widely expressed in goldfish tissues, including the brain and heart (59). The abundant expression of *APLN* in chicken brain, heart, and lung also highlights the important roles of apelin-APLNR1 axis in these tissues, such as the control of food intake and stress in the CNS and cardiovascular development or function, as demonstrated in fish and mammals (2, 60, 61). In addition, *cAPLN* mRNA is also weakly expressed in other tissues examined, including adipose tissue, kidneys, liver, muscle, and spleen. This finding also suggests an autocrine/paracrine action of apelin in these tissues, such as regulation of lipid metabolism, muscle glucose uptake, and spleen cytokine expression, as reported in mammals (2, 30, 62).

Since ELA is a novel ligand of APLNR identified recently (37), the information regarding its expression in vertebrates is extremely limited. In adult rats, *ELA* mRNA is exclusively expressed in kidneys and is capable of increasing water intake and urine flow rate (40). In humans, *ELA* is reported to be expressed only in adult kidneys, prostates, and embryonic stem cells (hECS) (37, 41). In zebrafish, *ELA* is transiently expressed during embryogenesis, which is believed to act as a developmental signal to control cardiovascular development (37, 38). In this study, *ELA* was detected to be widely expressed in adult chicken tissues, with a high mRNA level noted in the pancreas, kidneys, liver and spleen. The wide expression of *cELA* not only suggests that cELA may regulate kidney functions in chickens, such as diuresis previously demonstrated in rats, but also implies that cELA may play active roles in non-kidney tissues, including pancreas, liver, and spleen in a way analogous to apelin (2, 62).

Like *cAPLNR1, cAPLNR2* mRNA is widely expressed in chicken tissues, including the brain and heart. Similar wide tissue expression pattern of *APLNR2* has also been reported in adult bullfrogs (58). Considering that cAPLNR2 can only be activated by high concentrations of apelin-36 or ELA-32, it remains a mystery whether APLNR2 alone can play a substantial role in mediating actions of apelin/ELA in chickens.

In zebrafish, three *APLNRs* are widely, but differentially, expressed in adult tissues. Similarly, *APLN* is also widely expressed in zebrafish tissues (Figure 7). This is consistent with the finding in chickens, in which both *APLN* and *APLNRs* are expressed in diverse tissues, suggesting a broad spectrum of actions associated with the apelin-APLNR axis in both the CNS and peripheral tissues of non-mammalian vertebrates, as demonstrated in mammals (2, 30, 61, 62). It is of particular interest to note that *ELA* is predominantly expressed in zebrafish testes, suggesting that in addition to being a developmental signal in regulating cardiovascular development (37–39), *ELA* may act as an autocrine/paracrine signal to regulate testis functions, such as spermatogenesis or steroidogenesis at adult stage. In this study, we also noted that *APLNRs* (*APLNR2b, APLNR3a*) are abundantly expressed in zebrafish spleen (Figure 7). The high mRNA level of *APLNR* was also detected in the spleen of chickens (Figure 7) and humans (7), implying the importance of ALPNR signaling in vertebrate spleen, which warrants further investigation.

It is reported that *APLNR2s* and *ELA* are transiently and abundantly expressed at gastrulation stage (5.25–10 hpf) and regulate the movement of mesoendodermal cells, heart development, and angioblast migration (37–39). Unlike *APLNR2s* and *ELA, APLNR3a* is highly expressed during segmentation period (10–24 hpf) of zebrafish embryos. This finding, together with the expression of *APLN* (not *ELA*) at the later stage (Figure 8), suggests a yet-to-be-identified role of apelin-APLNR3a signaling in late embryogenesis of zebrafish.

In summary, we characterized two APLNRs in chickens and turtles, and three APLNRs in zebrafish. Functional study proved that chicken APLNR1 (and not APLNR2) can be activated by apelin-36 and ELA-32 potently, indicating that it is a major functional receptor common for both peptides in chickens. In sharp contrast, in turtles and zebrafish, APLNR2(s) can be activated by apelin-36 and ELA-32 potently, supporting the notion that it is the major functional receptor(s) common for both peptides, while APLNR1 is likely lost, or non-functional in these lineages. Strikingly, a novel apelin-specific receptor (APLNR3a/APLNR3b) was identified in zebrafish and tilapia. The identification and functional characterization of APLNRs in model vertebrates not only provides a clear molecular basis for interpreting the actions of APLNR in different classes of vertebrates, but also suggests a functional switch between APLNR1 and APLNR2/3 in mediating apelin and ELA actions during vertebrate evolution (Figure 10).

## Author's Note

The major content of this work was presented in the 11th International Symposium in Avian Endocrinology (Oct. 11–14, 2016, Niagara, Canada), p70 (Abstract).

## Author Contributions

JZ, YZ, CW, YiW, CF, JiL, WF, RY, and GZ conducted the experiments. JZ, JuL, and YaW designed and drafted the manuscript. All authors read and approved the final manuscript and joined the analysis and interpretation of data.

### Conflict of Interest Statement

The authors declare that the research was conducted in the absence of any commercial or financial relationships that could be construed as a potential conflict of interest.

## References

[1] O'Dowd BF Heiber M Chan A Heng HH Tsui LC Kennedy JL. A human gene that shows identity with the gene encoding the angiotensin receptor is located on chromosome 11. Gene (1993) 136:355–60. 10.1016/0378-1119(93)90495-O 8294032

[2] Pitkin SL Maguire JJ Bonner TI Davenport AP. International union of basic and clinical pharmacology. LXXIV. Apelin receptor nomenclature, distribution, pharmacology, and function. Pharmacol Rev. (2010) 62:331–42. 10.1124/pr.110.002949 20605969

[3] Tatemoto K Hosoya M Habata Y Fujii R Kakegawa T Zou MX. Isolation and characterization of a novel endogenous peptide ligand for the human APJ receptor. Biochem Biophys Res Commun. (1998) 251:471–6. 10.1006/bbrc.1998.9489 9792798

[4] Hosoya M Kawamata Y Fukusumi S Fujii R Habata Y Hinuma S. Molecular and functional characteristics of APJ tissue distribution of mRNA and interaction with the endogenous ligand apelin. J Biol Chem. (2000) 275:21061–7. 10.1074/jbc.M908417199 10777510

[5] O'Carroll AM Lolait SJ Harris LE Pope GR. The apelin receptor APJ: journey from an orphan to a multifaceted regulator of homeostasis. J Endocrinol. (2013) 219:R13–35. 10.1530/JOE-13-0227 23943882

[6] Kawamata Y Habata Y Fukusumi S Hosoya M Fujii R Hinuma S. Molecular properties of apelin: tissue distribution and receptor binding. Biochim Biophys Acta (2001) 1538:162–71. 10.1016/S0167-4889(00)00143-9 11336787

[7] Medhurst AD Jennings CA Robbins MJ Davis RP Ellis C Winborn KY. Pharmacological and immunohistochemical characterization of the APJ receptor and its endogenous ligand apelin. J Neurochem. (2003) 84:1162–72. 10.1046/j.1471-4159.2003.01587.x 12603839

[8] Masri B Lahlou H Mazarguil H Knibiehler B Audigier Y. Apelin (65–77) activates extracellular signal-regulated kinases via a PTX-sensitive G protein. Biochem Biophys Res Commun. (2002) 290:539–45. 10.1006/bbrc.2001.6230 11779205

[9] De Mota N Lenkei Z Llorens-Cortes C Cloning, pharmacological characterization and brain distribution of the rat apelin receptor. Neuroendocrinology (2000) 72:400–7. 10.1159/000054609 11146423

[10] O'Carroll AM Selby TL Palkovits M Lolait SJ. Distribution of mRNA encoding B78/apj, the rat homologue of the human APJ receptor, and its endogenous ligand apelin in brain and peripheral tissues. Biochim Biophys Acta (2000) 1492:72–80. 10.1016/S0167-4781(00)00072-5 11004481

[11] Reaux A Gallatz K Palkovits M Llorens-Cortes C. Distribution of apelin-synthesizing neurons in the adult rat brain. Neuroscience (2002) 113:653–62. 10.1016/S0306-4522(02)00192-6 12150785

[12] Kleinz MJ Davenport AP. Immunocytochemical localization of the endogenous vasoactive peptide apelin to human vascular and endocardial endothelial cells. Regul Pept. (2004) 118:119–25. 10.1016/j.regpep.2003.11.002 15003827

[13] Kleinz MJ Skepper JN Davenport AP. Immunocytochemical localisation of the apelin receptor, APJ, to human cardiomyocytes, vascular smooth muscle and endothelial cells. Regul Pept. (2005) 126:233–40. 10.1016/j.regpep.2004.10.019 15664671

[14] Kostopoulos CG Spiroglou SG Varakis JN Apostolakis E Papadaki HH. Adiponectin/T-cadherin and apelin/APJ expression in human arteries and periadventitial fat: implication of local adipokine signaling in atherosclerosis? Cardiovasc Pathol. (2014) 23:131–8. 10.1016/j.carpath.2014.02.003 24675084

[15] Charo DN Ho M Fajardo G Kawana M Kundu RK Sheikh AY. Endogenous regulation of cardiovascular function by apelin-APJ. Am J Physiol Heart Circ Physiol. (2009) 297:H1904–13. 10.1152/ajpheart.00686.2009 19767528 PMC2781363

[16] Kuba K Zhang L Imai Y Arab S Chen M Maekawa Y. Impaired heart contractility in Apelin gene–deficient mice associated with aging and pressure overload. Circul Res. (2007) 101:e32–42. 10.1161/CIRCRESAHA.107.158659 17673668

[17] Ishida J Hashimoto T Hashimoto Y Nishiwaki S Iguchi T Harada S. Regulatory roles for APJ, a seven-transmembrane receptor related to angiotensin-type 1 receptor in blood pressure *in vivo*. J Biol Chem. (2004) 279:26274–9. 10.1074/jbc.M404149200 15087458

[18] Tatemoto K Takayama K Zou M.-X. Kumaki I Zhang W Kumano K. The novel peptide apelin lowers blood pressure via a nitric oxide-dependent mechanism. Regul Pept. (2001) 99:87–92. 10.1016/S0167-0115(01)00236-1 11384769

[19] De Mota N Reaux-Le Goazigo A El Messari S Chartrel N Roesch D Dujardin C Apelin, a potent diuretic neuropeptide counteracting vasopressin actions through inhibition of vasopressin neuron activity and vasopressin release. Proc Natl Acad Sci USA. (2004) 101:10464–9. 10.1073/pnas.0403518101 15231996 PMC478592

[20] Tempel D de Boer M van Deel ED Haasdijk RA Duncker DJ Cheng C. Apelin enhances cardiac neovascularization after myocardial infarction by recruiting Aplnr+ circulating cells. Circul Res. (2012) 111:585–98. 10.1161/CIRCRESAHA.111.262097 22753078

[21] Newson MJ Roberts EM Pope GR Lolait SJ O'Carroll AM. The effects of apelin on hypothalamic–pituitary–adrenal axis neuroendocrine function are mediated through corticotrophin-releasing factor-and vasopressin-dependent mechanisms. J Endocrinol. (2009) 202:123–9. 10.1677/JOE-09-0093 19395447 PMC2695660

[22] Kang Y Kim J Anderson JP Wu J Gleim SR Kundu RK. Apelin-APJ signaling is a critical regulator of endothelial MEF2 activation in cardiovascular development. Circul Res. (2013) 113:22–31. 10.1161/CIRCRESAHA.113.301324 23603510 PMC3739451

[23] D'aniello C Lonardo E Iaconis S Guardiola O Liguoro AM Liguori GL. G protein-coupled receptor APJ and its ligand apelin act downstream of Cripto to specify embryonic stem cells toward the cardiac lineage through extracellular signal-regulated kinase/p70S6 kinase signaling pathway. Circul Res. (2009) 105:231–8. 10.1161/CIRCRESAHA.109.201186 19574549

[24] Paskaradevan S Scott IC The Aplnr GPCR regulates myocardial progenitor development via a novel cell-non-autonomous, Gαi/o protein-independent pathway. Biol Open (2012) 1:275–85. 10.1242/bio.2012380 23213418 PMC3507289

[25] Sunter D Hewson AK Dickson SL. Intracerebroventricular injection of apelin-13 reduces food intake in the rat. Neurosci Lett. (2003) 353:1–4. 10.1016/S0304-3940(03)00351-3 14642423

[26] Lv SY Yang YJ Qin YJ Mo JR Wang NB Wang YJ. Central apelin-13 inhibits food intake via the CRF receptor in mice. Peptides (2012) 33:132–8. 10.1016/j.peptides.2011.11.011 22108714

[27] Taheri S Murphy K Cohen M Sujkovic E Kennedy A Dhillo W. The effects of centrally administered apelin-13 on food intake, water intake and pituitary hormone release in rats. Biochem Biophys Res Commun. (2002) 291:1208–12. 10.1006/bbrc.2002.6575 11883945

[28] Lee DK Cheng R Nguyen T Fan T Kariyawasam AP Liu Y. Characterization of apelin, the ligand for the APJ receptor. J Neurochem. (2000) 74:34–41. 10.1046/j.1471-4159.2000.0740034.x 10617103

[29] Reaux-Le Goazigo A Alvear-Perez R Zizzari P Epelbaum J Bluet-Pajot MT Llorens-Cortes C Cellular localization of apelin and its receptor in the anterior pituitary: evidence for a direct stimulatory action of apelin on ACTH release. Am J Physiol Endocrinol Metabol. (2007) 292:E7–15. 10.1152/ajpendo.00521.2005 16896162

[30] Bertrand C Valet P Castan-Laurell I. Apelin and energy metabolism. Front Physiol. (2015) 6:115. 10.3389/fphys.2015.00115 25914650 PMC4392293

[31] Cheng B Chen J Bai B Xin Q. Neuroprotection of apelin and its signaling pathway. Peptides (2012) 37:171–3. 10.1016/j.peptides.2012.07.012 22820556

[32] Cox CM D'Agostino SL Miller MK Heimark RL Krieg PA. Apelin, the ligand for the endothelial G-protein-coupled receptor, APJ, is a potent angiogenic factor required for normal vascular development of the frog embryo. Dev Biol. (2006) 296:177–89. 10.1016/j.ydbio.2006.04.452 16750822

[33] Kälin RE Kretz MP Meyer AM Kispert A Heppner FL Brändli AW. Paracrine and autocrine mechanisms of apelin signaling govern embryonic and tumor angiogenesis. Dev Biol. (2007) 305:599–614. 10.1016/j.ydbio.2007.03.004 17412318

[34] Scott IC Masri B D'Amico LA Jin SW Jungblut B Wehman AM. The g protein-coupled receptor agtrl1b regulates early development of myocardial progenitors. Dev Cell (2007) 12:403–13. 10.1016/j.devcel.2007.01.012 17336906

[35] Zeng XX Wilm TP Sepich DS Solnica-Krezel L. Apelin and its receptor control heart field formation during zebrafish gastrulation. Dev Cell (2007) 12:391–402. 10.1016/j.devcel.2007.01.011 17336905

[36] Quertermous T. Apelin and its g protein-coupled receptor regulate cardiac development as well as cardiac function. Dev Cell (2007) 12:319–20. 10.1016/j.devcel.2007.02.005 17336895

[37] Chng SC Ho L Tian J Reversade B. ELABELA: a hormone essential for heart development signals via the apelin receptor. Dev Cell (2013) 27:672–80. 10.1016/j.devcel.2013.11.002 24316148

[38] Pauli A Norris ML Valen E Chew GL Gagnon JA Zimmerman S. Toddler: an embryonic signal that promotes cell movement via Apelin receptors. Science (2014) 343:1248636. 10.1126/science.1248636 24407481 PMC4107353

[39] Helker CS Schuermann A Pollmann C Chng SC Kiefer F Reversade B. The hormonal peptide Elabela guides angioblasts to the midline during vasculogenesis. Elife (2015) 4:e06726. 10.7554/eLife.06726 26017639 PMC4468421

[40] Deng C Chen H Yang N Feng Y Hsueh AJ. Apela regulates fluid homeostasis by binding to the APJ receptor to activate Gi signaling. J Biol Chem. (2015) 290:18261–8. 10.1074/jbc.M115.648238 25995451 PMC4513087

[41] Wang Z Yu D Wang M Wang Q Kouznetsova J Yang R. Elabela-apelin receptor signaling pathway is functional in mammalian systems. Sci Rep. (2015) 5:8170. 10.1038/srep08170 25639753 PMC4313117

[42] Zhang J Wan Y Fang C Chen J Ouyang W Li J. The orphan G protein-coupled receptor 25 (GPR25) is activated by Apelin and Apela in non-mammalian vertebrates. Biochem Biophys Res Commun. (2018) 501:408–14. 10.1016/j.bbrc.2018.04.229 29727602

[43] Ho L van Dijk M Chye STJ Messerschmidt DM Chng SC Ong S. ELABELA deficiency promotes preeclampsia and cardiovascular malformations in mice. Science (2017) 357:707–13. 10.1126/science.aam6607 28663440

[44] Freyer L Hsu CW Nowotschin S Pauli A Ishida J Kuba K. Loss of Apela peptide in mice causes low penetrance embryonic lethality and defects in early mesodermal derivatives. Cell Rep. (2017) 20:2116–30. 10.1016/j.celrep.2017.08.014 28854362 PMC5580402

[45] Ho L Tan SY Wee S Wu Y Tan SJ Ramakrishna NB. ELABELA is an endogenous growth factor that sustains hESC self-renewal via the PI3K/AKT pathway. Cell Stem Cell (2015) 17:435–47. 10.1016/j.stem.2015.08.010 26387754

[46] Huang G He C Meng F Li J Zhang J Wang Y. Glucagon-like peptide (GCGL) is a novel potential TSH-releasing factor (TRF) in Chickens: I) Evidence for its potent and specific action on stimulating TSH mRNA expression and secretion in the pituitary. Endocrinology (2014) 155:4568–80. 10.1210/en.2014-1331 25076122

[47] Cai G Mo C Huang L Li J Wang Y. Characterization of the two CART genes (CART1 and CART2) in chickens (*Gallus gallus*). PLoS ONE (2015) 10:e0127107. 10.1371/journal.pone.0127107 25992897 PMC4436185

[48] Meng F Huang G Gao S Li J Yan Z Wang Y. Identification of the receptors for somatostatin (SST) and cortistatin (CST) in chickens and investigation of the roles of cSST28, cSST14, and cCST14 in inhibiting cGHRH1–27NH2-induced growth hormone secretion in cultured chicken pituitary cells. Mol Cell Endocrinol. (2014) 384:83–95. 10.1016/j.mce.2014.01.001 24418361

[49] Wan YP Zhang JN Fang C Chen JN Li J Li J. Characterization of neuromedin U (NMU), neuromedin S (NMS) and their receptors (NMUR1, NMUR2) in chickens. Peptides (2018) 101:69–81. 10.1016/j.peptides.2017.12.022 29288685

[50] Mo C Huang L Cui L Lv C Lin D Song L. Characterization of NMB, GRP and their receptors (BRS3, NMBR and GRPR) in chickens. J Mol Endocrinol. (2017) 59:61–79. 10.1530/JME-17-0020 28500250

[51] Bu G Lin D Cui L Huang L Lv C Huang S. Characterization of neuropeptide B (NPB), neuropeptide W (NPW), and their receptors in chickens: evidence for NPW being a novel inhibitor of pituitary GH and prolactin secretion. Endocrinology (2016) 157:3562–76. 10.1210/en.2016-1141 27399877

[52] Tucker B Hepperle C Kortschak D Rainbird B Wells S Oates AC. Zebrafish Angiotensin II Receptor-like 1a (agtrl1a) is expressed in migrating hypoblast, vasculature, and in multiple embryonic epithelia. Gene Expr Patterns (2007) 7:258–65. 10.1016/j.modgep.2006.09.006 17085078

[53] Meyer A Van de Peer Y. From 2R to 3R: evidence for a fish-specific genome duplication (FSGD). Bioessays (2005) 27:937–45. 10.1002/bies.20293 16108068

[54] Nakatani Y Takeda H Kohara Y Morishita S. Reconstruction of the vertebrate ancestral genome reveals dynamic genome reorganization in early vertebrates. Genome Res. (2007) 17:1254–65. 10.1101/gr.6316407 17652425 PMC1950894

[55] Maguire JJ Kleinz MJ Pitkin SL Davenport AP. [Pyr1] apelin-13 identified as the predominant apelin isoform in the human heart: vasoactive mechanisms and inotropic action in disease. Hypertension (2009) 54:598–604. 10.1161/HYPERTENSIONAHA.109.134619 19597036

[56] Masri B Morin N Pedebernade L Knibiehler B Audigier Y. The apelin receptor is coupled to Gi1 or Gi2 protein and is differentially desensitized by apelin fragments. J Biol Chem. (2006) 281:18317–26. 10.1074/jbc.M600606200 16679320

[57] Chen X Bai B Tian Y Du H Chen J. Identification of serine 348 on the apelin receptor as a novel regulatory phosphorylation site in apelin-13-induced G protein-independent biased signaling. J Biol Chem. (2014) 289:31173–87. 10.1074/jbc.M114.574020 25271156 PMC4223320

[58] Moon MJ Moon JS Kim DK Hwang JI Lee JY Kim JI Cloning and activation of the bullfrog apelin receptor: Gi/o coupling and high affinity for [Pro1] apelin-13. Mol Cell Endocrinol. (2007) 277:51–60. 10.1016/j.mce.2007.07.008 17825479

[59] Volkoff H Wyatt JL. Apelin in goldfish (*Carassius auratus*): cloning, distribution and role in appetite regulation. Peptides (2009) 30:1434–40. 10.1016/j.peptides.2009.04.020 19427346

[60] Penney CC Volkoff H. Peripheral injections of cholecystokinin, apelin, ghrelin and orexin in cavefish (*Astyanax fasciatus mexicanus*): effects on feeding and on the brain expression levels of tyrosine hydroxylase, mechanistic target of rapamycin and appetite-related hormones. Gen Comp Endocrinol. (2014) 196:34–40. 10.1016/j.ygcen.2013.11.015 24287340

[61] Yang P Maguire JJ Davenport AP. Apelin, Elabela/Toddler, and biased agonists as novel therapeutic agents in the cardiovascular system. Trends Pharmacol Sci. (2015) 36:560–7. 10.1016/j.tips.2015.06.002 26143239 PMC4577653

[62] CarpénéCDrayCAttaneCValetPPortilloMChurrucaI. Expanding role for the apelin/APJ system in physiopathology. J Physiol Biochem. (2007) 63:358–73. 10.1007/BF0316576718457011

