# Supplementary material for: Characterization of the Apelin/Elabela Receptors (APLNR) in Chickens, Turtles, and Zebrafish: Identification of a Novel Apelin-Specific Receptor in Teleosts
Source: Front Endocrinol (Lausanne). 2018 Dec 13;9:756. doi: 10.3389/fendo.2018.00756 (PMC6315173; doi:10.3389/fendo.2018.00756)
Supplement: Supplementary file 1 [file Data_Sheet_1.doc]

**Supplementary Information**

**Title:**

**Characterization of the Apelin/Elabela Receptors (APLNR) in Chickens, Turtles and Zebrafish:** **Identification of a Novel Apelin-specific Receptor in Teleosts**

**Authors:**

Jiannan Zhang, Yawei Zhou, Chenlei Wu, Yiping Wan, Chao Fang, Jing Li, Wenqian Fang, Ran Yi, Guoqiang Zhu, Juan Li^*^, Yajun Wang^*^

**A**

**cAPLN**--1---GATGACCGGGCGGGCGCGGGGCCGGGCCGGGCTCTCACCGCCTACAAATGCGGCGATCCC-60--

**cAPLN**--61--GGAGCGGGGCCACCGCCTCCTGCGTGTCCGTGTCCCCGCGCGTCCGTGTCCCCGCGCCGG-120-

**cAPLN**--121-GCGGAGCATGGCGGCGCGCCGCTGGCTGCTGGCGCTGCTGCTGCTCTTCTGCCTCGCGCT-180-

-------------------M--A--A--R--R--W--L--L--A--L--L--L--L--F--C--L--A--L-

**(Exon-1)**

**cAPLN**--181-GAGCGCCGCGTCCGCGGGTCCGCTGGGAGCGGGGCTGGACGGGATGGATCCCGAGGACGG-240-

-------------S--A--A--S--A--G--P--L--G--A--G--L--D--G--M--D--P--E--D--G-

**(Exon-2)**

**cAPLN**--241-CCTCATCCGAACGCTGGTGCGGCCCCGAGGTGCGCGGCGTGGGAACGTGCGGCGGCCGGG-300-

-------------L--I--R--T--L--**V--R--P--R--G--A--R--R--G--N--V--R--R--P--G**-

**cAPLN**--301-AGGGTGGCGGAGGCTCCGCCGTCCCCGGCCACGGCTGTCCCATAAGGGCCCCATGCCCTT-360-

-----------**--G--W--R--R--L--R--R--P--R--P--R--L--S--H--K--G--P--M--P--F**-

**cAPLN**--361-CTGATGGAGGACCACCGCCAGCGCTCCGAGGATGCATCCCCACCGCCCGCTCTGCTCGTC-420-

-------------*-- **(Exon-3)**

**cAPLN**--421-CCTGAGCCCACCCGAGAGCTGGCGTCGCCTCCCTGCAGCTGTCCCTGGTGGCTCTGGCAC-480-

**cAPLN**--481-CGCGTCCTCCCAGTGCCCCGTGCCCTGATCCCGGTGTGCCAGGTCCAGGGGCTCTGCATA-540-

**cAPLN**--541-AAGCACTTTGTCCGGCGGAGGAGCTCTCCGTGGGGTCCCGTCCTGCCCCATCCCCTCCCG-600-

**cAPLN**--601-GAGGGGTCTATTTATTCCTGTGGTGCCGACCCCCTGTGAGCTGCCCTGTGGGGTTCCCAC-660-

**cAPLN**--661-CTCCAGGGCTCTGCAGGAAGCCACTGATGTACCCCAAAGGGGCCGCAGCGGTGCCGGGCC-720-

**cAPLN**--721-CTGCTGGTCACCGTGTCTGTCACGTCTGTAAAGAATTGTTCTGTGTAAATACATCTTTAT-780-

**cAPLN**--781-TATAAAGAGCTATGATGACAATAAAAAAAAAAAAAAAAAAAAAAAAAAAAAAAAAAAAAA-840-

**cAPLN**--841-AAAACTAAAAAAAAAAAAAAAAAAAAAAAAAAAAAAAA-878-

**B**

**cELA**--1---GCTATTTGCCCGTCTAGGAGAGGGTCTGCCGCTAGCATGTGACACGAATGAGGCGAGTGC-60--

**cELA**--61--CACGGACGCTTCTGTACACACGCGGACCGCAGGGATGAGGCTCCGGCGGCTGCTGTGCGT-120-

---------------------------------------------M--R--L--R--R--L--L--C--V-

**(Exon-1)**

**cELA**--121-CGTGTTCCTGCTCCTGGTCAGCCTGCTACCTGCCGCCGCGCAAAGACCGGCCAACCTGGC-180-

------------V--F--L--L--L--V--S--L--L--P--A--A--A-**-Q--R--P--A--N--L--A-**

**(Exon-2)**

**cELA**--181-CCTGCGCAGGAAGCTGCACCGACACAACTGCTCGCACCGGCGGTGCATGCCGCTCCACTC-240-

----------**--L--R--R--K--L--H--R--H--N--C--S--H--R--R--C--M--P--L--H--S-**

**cELA**--241-CCGCGTGCCCTTTCCCTGAGCGCCCGGCCCAGCTCGGCAAGCAATTTCGTAACGGGCTTT-300-

----------**--R--V--P--F--P-**-*-

**(Exon-3)**

**cELA**--301-TCAGTGTCTTAAAGGAGGAAGCTGCAACAACTGCACTGATAGAGAAGCTCATTCTAAGTA-360-

**cELA**--361-CTGCTTACCAACAGTTGACCTGGTGGAGCAACAGCAATCCTGTTTTGAGGGAGTCCATCT-420-

**cELA**--421-GAAATGAACCCTTTCAGTGGTCCTGTGTATCACATTCTGCATGACCTGGAACAAATGCCC-480-

**cELA**--481-ATGACTCATATCCTAGAAGCAGGGGGAAGGGAGAAACGGGGAAGGTGATTGGGGGGTGGG-540-

**cELA**--541-GGAGGTTGTACATGAAGCTTCCCTGGTTGAAGTGTATATTATTTGAATCTCAAAATGATC-600-

**cELA**--601-AGTAAAAAAAAAAAAAAAAAAAAAAAAAAA-630-

**Supplementary Figure 1.** **The nucleotide and amino acid sequences of (A) *cAPLN* (KX017222) and (B) *cELA* (KX017223)**. The mature peptide region of apelin (apelin-36) or ELA (ELA-32) is boxed. Both *APLN* and *ELA* genes consist of three exons including a non-coding exon (exon 3) at their 3’-UTR. The two arrows indicate the locations of two introns (introns 1 and 2).

**chAPLNR1**--1---MEETA--YTYTEGDNET--DCGYEEWGLSLALLPTIYLLVFLLGTAGNGLVLWTIFRGGRERRRSADTFIANLAAADLTFVVTLPLWAAYAWLGYHWPFG-96--

**slAPLNR1**--1---MEEQL--DWAAYGENLTG-DCEYEEWSPSLVLLPTIYLLVFLLGTIGNDLVLWTIFRGGQEKRRSADTFIANLAMADLTFVVTLPLWAAYAWLGYHWPFG-97--

**mAPLNR1-**--1---MEDDG--YNYYGADNQS--ECDYADWKPSGALIPAIYMLVFLLGTTGNGLVLWTVFRTSREKRRSADIFIASLAVADLTFVVTLPLWATYTYREFDWPFG-96--

**huAPLNR1**--1---MEEGGDFDNYYGADNQS--ECEYTDWKSSGALIPAIYMLVFLLGTTGNGLVLWTVFRSSREKRRSADIFIASLAVADLTFVVTLPLWATYTYRDYDWPFG-98--

**coAPLNR1**--1---MTPYLEKDNFSDGSNDSQIPCPFDEWEPSFALIPIIYFLIFILGLSGNGMVIWMVFH-MKEKRRSADTFIANLALADLTFVVSLPLWAVYTAQRYHWVFG-99--

**chAPLNR1**--97--TATCKVSSYLVFVNMYASVFCLTGLSFDRYLAIVRPLATAKLRSRVSGLLATVALWVLAALLALPALVLRQAATLGGDTKVTCYMDYGGLAALGTE-AAW-195-

**slAPLNR1**--98--SFTCKLSSYLIFVNMHASVFCLMGLSIDRYLAIVRPMANAKLRWRASGLVATIILWTLAAILALPAMILRQAAVLLGETKVTCYMDYSGVVANGTE-GAW-196-

**mAPLNR1-**--97--TFSCKLSSYLIFVNMYASVFCLTGLSFDRYLAIVRPVANARLRLRVSGAVATAVLWVLAALLAVPVMVFRSTDASENGTKIQCYMDYSMVATSNSE-WAW-195-

**huAPLNR1**--99--TFFCKLSSYLIFVNMYASVFCLTGLSFDRYLAIVRPVANARLRLRVSGAVATAVLWVLAALLAMPVMVLRTTGDLENTTKVQCYMDYSMVATVSSE-WAW-197-

**coAPLNR1**--100-TFLCKLSSYLIYINMYASVFCLTCLSFDRYLAIVRSMARSHLRSKTSSFMAIIVVWVLAAILALPALVFRSTSG--HEAPNSCFMDFSFAVEEEEEEIFW-197-

**chAPLNR1**--196-EVGLGLSSTLLGFVAPFAVMLTCYFFIARTVATHFRRERAEGPRKRKRLLTIIAVLVAAFGGCWLPFHLVKTLYILMELEALPWSCSLYNFLNNFHPYCT-295-

**slAPLNR1**--197-EVGLGLSSTMLGFVVPFVVMLTCYFFIARTIADHFRKERGEELRKRKRLLSIIIVLVATFAFCWLPYHLVKTIYVLMDLEVMPWSCGFHAFLNNLHPYCT-296-

**mAPLNR1-**--196-EVGLGVSSTAVGFVVPFTIMLTCYFFIAQTIAGHFRKERIEGLRKRRRLLSIIVVLVVTFALCWMPYHLVKTLYMLG--SLLHWPCDFDIFLMNVFPYCT-293-

**huAPLNR1**--198-EVGLGVSSTTVGFVVPFTIMLTCYFFIAQTIAGHFRKERIEGLRKRRRLLSIIVVLVVTFALCWMPYHLVKTLYMLG--SLLHWPCDFDLFLMNIFPYCT-295-

**coAPLNR1**--198-TAGLGISYTVLGFVLPFCIMTTCYFAIGRTVAQHFQKHK-EDLRKR-RLLTIIATLVATFAACWLPFHLVSTMYNLMYLDVIPFSCGLERFLMIVYPYAT-295-

**chAPLNR1**--296-GIAYINSCLNPFLYAFFDPHFRRACAALLCCHP-------PGPGAERSGSFSSGHSHPPG---GKGALAMGGKLDPATQETLFRT-----370-

**slAPLNR1**--297-CVAYINSCLNPFLYAFFDPRFRQACAAVLCCSRG----RWPGSSKDKSASYSSSHSQNLQ---GKGGELAQEKLGPGRQETLLRG-----374-

**mAPLNR1-**--294-CISYVNSCLNPFLYAFFDPRFRQACTSMLCCDQSGCKGTPHSSSAEKSASYSSGHSQGPGPNMGKGGEQMHEKSIPYSQETLVD------377-

**huAPLNR1**--296-CISYVNSCLNPFLYAFFDPRFRQACTSMLCCGQSRCAGTSHSSSGEKSASYSSGHSQGPGPNMGKGGEQMHEKSIPYSQETLVVD-----380-

**coAPLNR1**--296-CLAYINSCVNPFLYAFFDLRFRAQCLDILSCGR------LWSALQDKMMNFSSSNS-------------QNSKCDPQTLPSKV-------359-

**TMD1**

**TMD2**

**TMD3**

**TMD4**

**TMD5**

**TMD6**

**TMD7**

**(A) APLNR1**

**chAPLNR2-**--1-------MEYAEADYYYGEAEEEEEGNGTACAWQADWEASFALLPALYALVFVLGLAGNALVLLTVWRGPRAKRRSADAYIGNLALADLAFVATLPLWAAYTA-96--

**slAPLNR2-**--1----MEDAVGGEPDY--YYGEGNESGPGAQCEWPADWEVSFSLLPVLYMLVFVLGLSGNGLVIFTVWRGPRAKRRSADTYIGNLALADLAFVVTLPLWAAYTA-97--

**xtAPLNR2-**--1---MATDEFSSSTTPSYDYYDYTNESGLPPCDETD-WDLSYSLLPVFYMIVFVLGLSGNGVVIFTVWK-AKPKRRSADTYIGNLALADLAFVVTLPLWATYTA-98--

**coAPLNR2-**--1--------MNMNENYCGEYCEDNFTDS---CDYTD-WAISYSLIPVLYMLVFIFGLSGNGVVLFTVWK-TKLKRRSADIYIGNLALADLAFVITLPLWAVYTA-90--

**zfAPLNR2a**--1----MEPTPEYTETY--DYYDTGYNDSG--CDYSE-WEPSYSLIPVLYMLIFILGLSGNGVVIFTVWR-AKSKRRAADVYIGNLALADLTFVITLPLWAVYTA-93--

**zfAPLNR2b**--1----MNAMDNMTADYSPDYFDDAVNSSM--CEYDE-WEPSYSLIPVLYMLIFILGLTGNGVVIFTVWR-AQSKRRAADVYIGNLALADLTFVVTLPLWAVYTA-95--

**chAPLNR2-**--97--LGYHWPFGTATCKVSSYLVLLNMYASAFCLGGLSAERYRAVLRAAPPPPRAASARLRRPAALGPLAALWAAAAAAALPALLLREARRD-ARNRTLCDLRL-195-

**slAPLNR2-**--98--LRFHWPFGSALCKLSSYLVLLNMFASVFCLGSLSFERYLAIVRPLP----RSRPVRRRAAALLPLAALWLLAGLLALPALLLRDTQPGPADNLTVCDMDF-193-

**xtAPLNR2-**--99--LGFHWPFGSALCKLSSYLVLLNMFASVFCLTCLSFDRYLAIVHSLS----SAK-LRSRSSILVSLAVIWLFSGLLALPSLILRDTRVE--GNNTICDLDF-191-

**coAPLNR2-**--91--LGYHWPFGSALCKISSYLVLVNMYASIFCLTCLSFDRYLAIVHSLS----SSR-LRSRNTILLSLAITWILSGQLALPALILRTTIEK--DNVTFCDVDF-183-

**zfAPLNR2a**--94--LGYHWPFGVALCKISSYVVLVNMYASVFCLTCLSFDRYLAIVHSLS----SGR-LRSRATMLASLGAIWFLSCLLAVPTLLFRTTVDDTGSNRTTCAMDF-188-

**zfAPLNR2b**--96--LGYHWPFGVALCKISSYVVLLNMYASVFCLTCLSLDRYMAIVHSLT----STQ-LRTRGHMRASLTAIWLLSGVLAAPTLLFRTTVYDVETNRTSCAMDF-190-

**chAPLNR2-**--196-GGEGGGAERA---AAALSLGTTALGFAAPLLLMAVCYCCVGSAVRRHLRPRRAEAAARRRLLRLIAALVGVFAGCWLPFHLLKSLFVLAAAGLLELPCAL-292-

**slAPLNR2-**--194-SGVAS-AQSERYWRGALGLGTTALGFLLPLLLMTLFYCCIGATVSRHFQHLRKE-QEKRRLLRIIATLVVVFALCWLPFHLLKSLYVLSELELLELPCAF-291-

**xtAPLNR2-**--192-SGVSS-KENENFWIGGLSILTTVPGFLLPLLLMTIFYCFIGGKVTMHFQNLKKEEQKKKRLLKIIITLVVVFAICWLPFHILKTIHFLDLMGFLELSCST-290-

**coAPLNR2-**--184-SGLAE-PQNEHFWIVGLSMTTTFLGFFLPFLLMTIFYCFIGTTVTRHFQNLRKEDQKKKRLLKIIITLVVIFAICWLPFHVLKTIDAIAWLEIFPISCSF-282-

**zfAPLNR2a**--189-SLVTLNQDHESLWIAGLSLSSSALGFLLPFLAMTVCYCFIGCTVTRHFSHLRKEDQKKRRLLKIITTLVVVFAFCWTPFHVLKSMDALSYLDLAPNSCGF-288-

**zfAPLNR2b**--191-NLVVSQPGQETYWIAGLSISSTALGFLIPLLAMMVCYGFIGCTVTRHFNSLRKEDQRKRRLLKIITTLVVVFAACWMPFHVVKTMDALSYLNLAPDSCTF-290-

**chAPLNR2-**--293-LGLISRLHPYATCLAYLNSCLNPLLYAFLDGRFRAQCRALLGLRGARPP--PAASSTPSAPTQRSELPSSGGTKV-365-

**slAPLNR2-**--292-LGLIVLLHPYATCLAYINSCLNPFLYAFFDLRFRAQCRLLLALRPALRGPAGSGSSTLSAQTQKSELHS-LATKV-365-

**xtAPLNR2-**--291-QNIIVSLHPYATCLAYINSCLNPFLYAFFDLRFRSQCFFFFGFKKALQGHLSNTSSSLSAQTQKSEIHS-LATKV-364-

**coAPLNR2-**--283-QRFILLAHPYATCLAYVNSCLNPFLYAFFDLRFRSQCLCFLRLKKAVHGQISSVSSTMSGQTQKSEIQS-LATKV-356-

**zfAPLNR2a**--289-LHFLLLAHPYATCLAYANSCLNPFLYAFFDLRFRSQCLCLLNLKKAMHGHMSSMSSTLSAQTQKSEVQS-LATKV-362-

**zfAPLNR2b**--291-LNLLLLAHPYATCLAYVNSCLNPLLYAFFDLRFRSQCLCLLNLKKALH---ASPASSLSSQ--KTEAQS-LATKV-359-

**TMD1**

**TMD2**

**TMD3**

**TMD4**

**TMD5**

**TMD6**

**TMD7**

**(B) APLNR2**

**Supplementary Figure 2. Alignment of APLNR1/2 of chicken and other species. (A)** Alignment of chicken APLNR1 (chAPLNR1, KU887746) with that of red-eared sliders (slAPLNR1, KU887748), mice (mAPLNR, NP_035914), humans (huAPLNR1, NP_005152), and coelacanths (coAPLNR1, XP_005999317). (**B**) Alignment of chicken APLNR2 (chAPLNR2, KU887747) with that of red-eared sliders (slAPLNR2, KU887749), *Xenopus tropicalis* (xtAPLNR2, NP_001027492), coelacanths (coAPLNR2, XP_005990330), and zebrafish (zfAPLNR2a: NP_001068573; zfAPLNR2b; NP_001025368).

**zfAPLNR3a** 1 -----MSGSLPFP----SPSPFPSCDYTEWSPTWVLIPSVYLLVFVVGSLGNGLVLWVYLDRQARGRGRIGSGSKSSQTPD--SPPCPSPTSTRTVTESL 89

**cpAPLNR3a** 1 -----MSGSLPFP----SPSPFPSCDYKEWSATWVLIPSVYLLVFVVGSLGNGLVLWVYLDRRARGRGRTGSGSKSSQTTD--SPPCPSPTSSRTVTESL 89

**trAPLNR3a** 1 MSEFSMSELELFSA---SPPLLPQCDYSEWPATRVVIPAVYLLAFVAGTLGNGLVLWVYLDR--HGKGRRNSNSEQLQGP---STGSPSLTS-RSVTESL 91

**piAPLNR3a** 1 --------------------------YSEWRPTWVIIPSVYVLVFVLGSLGNGLVLWAYLDRPEGKPAGMAACTRRRLS-------PKLPGSSRSLTDLL 67

**saAPLNR3a** 1 -----MSDSELLPSASPSPPPLHQCDYREWRPTWVIIPSVYLLVFVLGSLGNGLVLWSYLDRPEGKPAGVGTCTKRRLQESGRSSTPKLPDSSRSLTDSL 95

**zfAPLNR3a** 90 IISLALADLAFVMTLPLWAAYTALGYHWPFGQVLCQTSSYIVALNMYASVFSLTGLSVERYCVITRQRGNGSKQRTSSTRARWIVGIVWLAAGVLALPAL 189

**cpAPLNR3a** 90 IASLALADLAFVMTLPLWAAYTALGFHWPFGHVLCQVSSYIVALNMYASVFSLTGLSVERYCVITRKYGSGSKQGRSTTRAKWIVGSVWLAAGILALPAL 189

**trAPLNR3a** 92 IASLALADLAFVMTLPLWAAYTALDYHWPFGGALCRASSYLVALNMYASVFSLTALSVERYCVITRSNSNNSMQSRATSRACWVVGSMWVAAFILALPAL 191

**piAPLNR3a** 68 IASLAAADLAFVITLPLWAAYTALDYHWPFSKPLCQVSSYLVVLNMYASVFSLTVLSIERYWVIAGRRRSDRTQGSGVCRAAWVVGSVWLVAGVLALPAL 167

**saAPLNR3a** 96 IASLAAADLAFVVTLPLWAAHTALDYHWPFGKSLCQVSSYLVALNMYASVFSLTVLSMERYWVIAGRRRSSRAQGSGVCRAAWVVGSVWVVAGILALPAL 195

**zfAPLNR3a** 190 LLRTVREPDL-ESGDDG----DWQAGQSSS------CLCDMDYSSLISSDLDPAEAEQAELMWSAALGLKSTLLGFLLPLVVLLVCYGWLGRLLSRHFSL 278

**cpAPLNR3a** 190 LLRTVREVNL-EEGDDG----DGEDGHVSS------CLCDMDYSSLISSELDPAASEQVELMWSAALGLKSTLLGFLLPLVVLLLCYGWLGRLLSRHFSL 278

**trAPLNR3a** 192 LLRAVRKVHL-ETGDSDSDWLDEEDGSGNSEGGTLVCLCDMDYSSLVSPDLDPTAREHAELIWSAALGLKSTILGFVLPLIVLLLCYCSLGRLLFHHFNL 290

**piAPLNR3a** 168 LLRTVRLVDVGEEYEDD----EEEEHT-----PLFISSCMMDYNGLISPELGDDDRIRAELLWTAALGLKSTLVGFLLPMVTLLICYCSLGRLLSRHFGQ 258

**saAPLNR3a** 196 LLRTVREVDVGVEYEEDWQTAEEEEHSDHDHMPMFISSCMMDYSGLISAELEEDDREQAELLWTAALGLKSTLVGFLLPLIILLICYCSLGRLLSRHFGQ 295

**zfAPLNR3a** 279 GLRPDQKRQRRLLRIIITLVLAFFLCWLPFHTNKTLSAMVELGILPFSCRFDQWLVAAHPYSICLGYVNSCLNPLLYACCDPAFRKRCSGLLLCLWVKCK 378

**cpAPLNR3a** 279 GPRPDHTRQRRLLRIIITLVLAFFLCWLPFHTNKTLSAMVELGILPFSCSFDQWLVAAHPYSICLGYVNSCLNPLLYACCDPAFRKHCSGLLVCARVKCR 378

**trAPLNR3a** 291 GPRPDRQRQRRLLRVIVTLVLAFFLCWLPFHVNKTLSALMELDLLPYSCGFDRWLVAAHPYAICLGYVNSCLNPLLYACCDPAFRRRCRGMVRYSWRSSA 390

**piAPLNR3a** 259 GPRPDRRRQRHLLRVIATLVLAFFLCWLPFHANKTLSMLMDLKMLPYSCPFDQMLVAAHPYATCLGYVNSCLNPLLYACCDVAFRKRCRALLQRGECRGY 358

**saAPLNR3a** 296 GPRPDRRRQRRLLRVIVTLVLAFFLCWLPFHANKTLSMLVDLELLPYSCPFDRTLVAAHPYATCLGYVNSCLNPLLYACCDVAFRKRCRALLQWLWCRGR 395

**zfAPLNR3a** 379 GQR-----AEEEKQQ-HQSSAVPSGTHEVTVSKDEQ----------------------------- 408

**cpAPLNR3a** 379 DQK-----GGKENEH-HKSSPIPSGTHEVTVSKEDQ----------------------------- 408

**trAPLNR3a** 391 RRG-----GEETRALPSKSSAMPSGTKEEVEEDEECGRVLEGEGGKEREVNDDKAICKDEYFERL 450

**piAPLNR3a** 359 WGRPEAATEADNREQVSRSLVILSGIQGETGNGIG----------------DDSRNGRG------ 401

**saAPLNR3a** 396 CGSPMTVDDDDNREQASRSSAIPSGTRKETGDGIEG--------------KDDVRHGRD------ 440

**TMD1**

**TMD2**

**TMD3**

**TMD4**

**TMD5**

**TMD6**

**TMD7**

**(A) APLNR3a**

**TiAPLNR3b** 1 -----MSEPAYLASPAPTNPTLCDYSDWSPSFFIIPSVYLLAFLVGCPGNSLVLWAYLDRVEGRRR--RDRKPAEDGQFCCSNIFRSSQQHINNSG---- 89

**mbAPLNR3b** 1 -----MSEPAYLASPAPTNPPLCDYSDWSPSFFIIPSVYLLAFLVGCPGNSLVLWAYLDRVKGRRR--RDRKPAEDGQFCCSNIFRSSQQHINNTG---- 89

**mdAPLNR3b** 1 -----MSDQDVPSSPSPTPFLLCDYSDWSPSLFIIPSVYLLAFLLGCLGNGLVLWAYLDRSEGRQTGAWGKQKREVRQLCCS--FRTRIKHAPHGC---- 89

**FuAPLNR3b** 1 METHTWDSVSPSSSPSPPSFSHCDYSDWSPSLTIIPSVYLLAFFVGCLGNSLVLWAYLDRPERRRM--RGRDLSGTGKLCFTGVFRKGQQTVRGTDGPHH 98

**TiAPLNR3b** 89 ----KVQSSNCGFSSRQSYRTSSHSSTSSSCSPSISRPSRSLTDSLIASLALADLCFLVTLPLWAVYTAMGYHWPFGQPLCQISSFLTALNMYASVFSLS 185

**mbAPLNR3b** 89 ----KFQSSNCGFSSRQSYRTSSHSSTSSSCSPSISRPSCSLTDSLIASLALADLCFLVTLPLWAVYTAMGYHWPFGQPLCQISSFLTALNMYASVFSLS 185

**mdAPLNR3b** 89 --FCPTNRSNVTDSSGQSSRPPCPSSNTH--PPLIPRPSRSLTDSLIASLALADLCFLVTLPLWAVYTAMGYHWIFGQALCQISSFLTALNMYASVFSLS 185

**FuAPLNR3b** 99 GSHPTKNKGNGGSLSGQTPKVSSHSSTTS-YPPSIPRSSRSLTDSLIASLALADLCFLVTLPLWAVYTAMGYHWPFGQPLCQVSSFLTALNMYASVFSLS 197

**TiAPLNR3b** 186 MLSVERYWVLTGRRHSSHHAPRSCPNKALWILGGMWVLAGILALPGLLLRSVREVELDPEYEDDWQLEPADS--RSVFLSCQMDYSMLTGAELEEEEKNR 283

**mbAPLNR3b** 186 MLSVERYWVLTRHRHSSHHAPRSCPSKALWILGGMWVLAGILALPGLLLRSVREVELDPEYEDDWPLEPADS--RSVFLSCQMDYSMLTGAELEEEEKNR 283

**mdAPLNR3b** 186 MLSLERYWILTGRQSSSQQPPQSWPSRAFWVLGGVWAVAGVLALPGLLLRSVREIELELDPKNSQQLDSRNA-----ILSCQMDYSIMIGSELDDSEKER 280

**FuAPLNR3b** 198 MLSVERYWVLTGRRPASHHAAQRFPSRAVWVLGGVWVLAGVLALPGLLLRSVREVEVE--SEDDWLIDPAHPDPGPVFLSCQMDYSMLIRTDLEEAEREK 295

**TiAPLNR3b** 284 TEMWWAAALSIKSTLLGFLLPLVILLVCYCSLVQLLSRHFGQGPRPDRRRQRRLLRVIVTLVMAFFLCWLPLHVNKTVSLLLEFGFVPYSCSLDQILLAA 383

**mbAPLNR3b** 284 TEMWWAAALSIKSTLIGFLLPFVILLVCYCSLVQLLSRHFGRGPRPDRRRQRRLLRVIVTLVMAFFLCWLPLHVNKTVSLLLEFGFVPYSCSLDQILLAA 383

**mdAPLNR3b** 281 MEMLWASALSIKSTLFGFLLPFIILLVCYCSLAQLLSRHFGRGPGTDRKRQRRLLRVIVTLVLAFFLCWLPLHVNKTMAMLLEFGFVAYSCSLDQSLLAA 380

**FuAPLNR3b** 296 AELWWAAALSLKSTLIGFLLPLVILLVCYCSLAQLLSRHFGKGPRPDRKRQRRLLKVIVTLVMAFFLCWLPLHVNKTLSMLLEFGLVPYSCPLDQLLLGA 395

**TiAPLNR3b** 384 HPYVTCLAYLNSCLNPLLYAACDPSFRKRCKGAFLVLCRMKSKGAKRKEGNEAEGVEEKGEQSSAFPMRTQEETADRTEEEQEGGVREMGVATPEELKKS 483

**mbAPLNR3b** 384 HPYVTCLAYLNSCLNPLLYAACDPSFRKRCKGAFLVLCRMKRKGAKGKEEHEAEGVEEKGEQSSAFPMRTQEETADRTEEEQEGGVREMGVATPEELKKS 483

**mdAPLNR3b** 381 HPYVTCLAYINSCLNPFLYAACDPSFRRRCKGALLTLCGICRGRGETKASKIDE-PDGKEDGSSTFPMRTQDTHR-------------LEDGEGEEMVLA 466

**FuAPLNR3b** 396 HPYVTCLAYLNSCLNPLLYAACDPSFRKRCRGAIVMLKWDN---------------------------------------------------NPPQM--- 441

**TiAPLNR3b** 484 RYQGAMFSDN 493

**mbAPLNR3b** 484 RYQGAMFSDN 493

**mdAPLNR3b** 467 VAEVAF---- 472

**FuAPLNR3b** 441 ---------- 441

**TMD1**

**TMD2**

**TMD3**

**TMD4**

**TMD5**

**TMD6**

**TMD7**

**(B) APLNR3b**

**Supplementary Figure 3.** **Alignment of APLNR3a/3b of zebrafish/tilapia and other teleost fishes.** (**A**) Alignment of zebrafish APLNR3a (zfAPLNR3a, KU887750) with that of common carp (cpAPLNR3a, KTG31429), Mexican tetra (trAPLNR3a: XP_007245029), northern pike (piAPLNR3a: XP_010880601), Atlantic salmon (XP_014030254). (**B**) Alignment of tilapia APLNR3b (TiAPLNR3b: KU887751) with that of zebra mbuna (mbAPLNR3b, XP_004563916), Japanese medaka (mdAPLNR3b, XP_011474208) and fugu (FuAPLNR3b, XP_011605916). Horizontal lines indicates the seven transmembrane domains (TMD1-7).

**Opposum-APLNR2-**--1---DEDYYYYYDYYNGSGGPACGGGGGEE--AEDWELSFWLLPLLYSLVFLLGLAANGAVLATVWGAPGGAGRRRASAAYVGH-78--

**Quail-APLNR2---**--1-----MDYAEADYYYGEAEEEE-GNGTACSWQADWEASFALLPALYALVFVLGLAGNALVLLTVWRGP--RSKRRSADAYIGN-75--

**Chicken-APLNR2-**--1-----MEYAEADYYYGEAEEEEEGNGTACAWQADWEASFALLPALYALVFVLGLAGNALVLLTVWRGP--RAKRRSADAYIGN-76--

**Opposum-APLNR2-**--79--LALADLALVAPLPLWAAYTALRFHWPFGAALCKGSSYVVLLAMFASAFCLAGLSFERYVAVGRSLSPGRPCARRGGPGPS-158-

**Quail-APLNR2---**--76--LALADLTFVVTLPLWAAYTALRFHWPFGAALCKLSSYLVLLNMFASAFCLGGLSAERYRAVLRAAPPP--------PRAA-147-

**Chicken-APLNR2-**--77--LALADLAFVATLPLWAAYTALRFHWPFGAALCKLSSYLVLLNMFASAFCLGGLSAERYRAVLRAAPPP--------PRAA-148-

**Opposum-APLNR2-**--159-PSPNPGPWAAAAPGALWLLAAVLALPALLLRATRSRPPANLTVCDLDLSGVAAAPEHRAVWLGALSLATTLLGFALPAVL-238-

**Quail-APLNR2---**--148-SARVRRPAALGPLAALWAAAAAAALPALLLREAR-RDARNRTLCDLRLGGEGGGAERAAA---ALSLGTTALGFAAPLLL-223-

**Chicken-APLNR2-**--149-SARLRRPAALGPLAALWAAAAAAALPALLLREAR-RDARNRTLCDLRLGGEGGGAERAAA---ALSLGTTALGFAAPLLL-224-

**Opposum-APLNR2-**--239-MGAFYCAIGRAVSRHLGVAAAGVRGARGRDRRRRLLRLLAGLVAVFTLCWLPFHVLKSLYVLSWLGLLALPCAAQALLVR-318-

**Quail-APLNR2---**--224-MAVCYCCVGSAVRRHLRPRRAEAA------ARRRLLRLIAALVGVFAGCWLPFHLLKSLFVLAAAGLLELPCALLGLISR-297-

**Chicken-APLNR2-**--225-MAVCYCCVGSAVRRHLRPRRAEAA------ARRRLLRLIAALVGVFAGCWLPFHLLKSLFVLAAAGLLELPCALLGLISR-298-

**Opposum-APLNR2-**--319-LHPYATCLAYVNSCLNPLLYAFLDHRFRAQCRRRLRSLLPCRRKGSRAGLAVDAADAAGPPAGEPAAPASSSASAASGPT-398-

**Quail-APLNR2---**--298-LHPYCTGIAYINSCLNPFLYAFLDGRFRAQCR----ALLGLR--GSRP-----------PPA---------ASSTLSAPT-351-

**Chicken-APLNR2-**--299-LHPYATCLAYLNSCLNPLLYAFLDGRFRAQCR----ALLGLR--GARP-----------PPA---------ASSTPSAPT-352-

**Opposum-APLNR2-**--399-QRSELAS-LATKV-410-

**Quail-APLNR2---**--352-QRSELPSSGGTKV-364-

**Chicken-APLNR2-**--353-QRSELPSSGGTKV-365-

**TMD1**

**TMD2**

**TMD3**

**TMD4**

**TMD5**

**TMD6**

**TMD7**

**Supplementary Figure 4.** **Alignment of opossum APLNR2 with quail/chicken APLNR2**. Partial amino acid sequence (XP_007490629) of opossum APLNR2 was predicted based on its genomic sequence (chromosome 3). Since opossum APLNR2 shows a low degree of identity (40%) with chicken APLNR2 (KU887747) or quail APLNR2 (XP_015733485), therefore it remains to be clarified whether it is functional. Although *APLNR2* does exist in the opposum, synteny analysis revealed that *APLNR2* is highly likely lost in most mammalian species, including humans and mice.

**Supplementary Table 1.** Amino acid sequence identity of APLNR1-3 among 5 vertebrate species including humans (h), chickens (c), red-eared sliders (tu), zebrafish (zf) and Nile tilapia (ti).


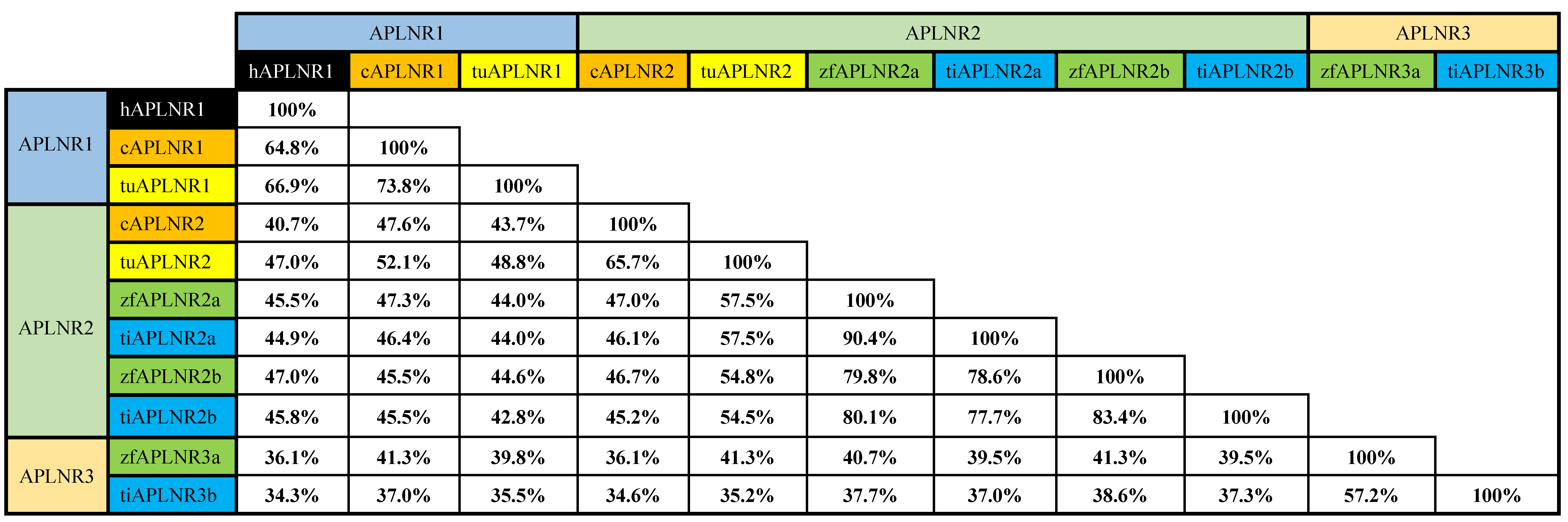


**Supplementary Table 2.** Primers used in this studya

| Gene | **Sense/antisense** | **Primer sequence (5’- to- 3’)** | **Size (bp)** |
| --- | --- | --- | --- |
| *Primers for rapid amplification of 5’-ends (5’-RACE)* | | | |
| *cAPLN* | Antisense | TGCAGAGCCCCTGGACCTGGCAC |  |
|  | Antisense | GTGACAGACACGGTGACCAGCAG |  |
| *cELA* | Antisense | ACACAGCGGTTCCCGTCAATAGGCG |  |
|  | antisense | GCACTCGCCTCACCCGTGTCACATG |  |
| *cAPLNR1* | antisense | AGGTTGGCGATGAAGGTGTCGGCTG |  |
|  | antisense | CGCCCCTGAAGATGGTCCAGAGCAC |  |
| *cAPLNR2* | antisense | CGGAGGCGAACATGTTGAGCAGCAC |  |
|  | antisense | CCAGCACGAAGACCAGCGCGTAGAG |  |
| *Primers for rapid amplification of 3’-ends (3’-RACE)* | | | |
| *cAPLN* | sense | GGCCTCATCCGAACGCTGGTGCG |  |
|  | sense | GACCACCGCCAGCGCTCCGAGGA |  |
| *cELA* | sense | CAGCTCGGCAAGCAATTTCGTAACG |  |
|  | sense | CTGGTGGAGCCACAGCAATCCTGTT |  |
| *Primers for constructing expression plasmids^b^* | | | |
| *hAPLNR1* | sense | CCGGAATTCAGCATGGAGGAAGGTGGTG | 1148 |
|  | antisense | CCGGAATTCCTAGTCAACCACAAGGGTC |  |
| *cAPLNR1* | sense | CCGGAATTCGGCATGGAGGAGACGGCGTAC | 1118 |
|  | antisense | CCGGAATTCTTTAGGTGCGGAAGAGCGTC |  |
| *cAPLNR2* | sense | CCGGAATTCGGGGCGATGGAGTACGCGGAG | 1176 |
|  | antisense | CCGGAATTCGCCGCGACGGGACGAAGAAG |  |
| *zfAPLNR2a* | sense | CCGGAATTCCAAATGGAGCCAACGTC | 1242 |
|  | antisense | CCGGAATTCATACTCGCATCCACTCATC |  |
| *zfAPLNR2b* | sense | CCGGAATTCAAGGAATGAATGCCATGGAC | 1121 |
|  | antisense | CCGGAATTCTTACCCCAATTCTGCGTCAC |  |
| *zfAPLNR3a* | sense | CCGGAATTCTGTCAAATGTCCGGGTCTCTG | 1278 |
|  | antisense | CCGGAATTCACCCAGCACGGTCCATTGAG |  |
| *tiAPLNR3b* | sense | CCGGAATTCAGAGTGATGTCAGAACCAGC | 1514 |
|  | antisense | CCGGAATTCGGTCATTACATAGGGTTAGG |  |
| *tuAPLNR1* | sense | CCGGAATTCGAGGCATGGAGGAGCAGCTG | 1141 |
|  | antisense | CCGGAATTCTCCCCCTTCTCTTAGCCTCG |  |
| *tuAPLNR2* | sense | CCGGAATTCAGTGCGGGGCCATGGAGGAC | 1119 |
|  | antisense | CCGGAATTCGCCGAGCGAGCTACACCTTG |  |
| *Primers for quantitative Real-time PCR assay* | | | |
| *cAPLN* | sense | CCCATGCCCTTCTGATGGAG | 193 |
|  | antisense | TTTATGCAGAGCCCCTGGAC |  |
| *cELA* | sense | GCTCCTGGTCAGCCTGCTAC | 91 |
|  | antisense | CCGGTGCGAGCAGTTGTGTC |  |
| *cAPLNR1* | sense | CTCGGGCAGCTTCTCCTC | 103 |
|  | antisense | GCGGAAGAGCGTCTCCTG |  |
| *cAPLNR2* | sense | GCGGAGGCGGATTATTAC | 126 |
|  | antisense | GAAGACCAGCGCGTAGAG |  |
| *zfAPLN* | sense | TCTCCCTCCCATCCACACAC | 173 |
|  | antisense | TTGCTATGCTCGGTGGAGGC |  |
| *zfELA* | sense | ACCAAACCACCCTGAGCATC | 165 |
|  | antisense | AGCGTTTCTTCGGGCAGTTG |  |
| *zfAPLNR2a* | sense | GCCGTCTTACTCTCTCATTC | 76 |
|  | antisense | ACAGCCCACAGCGGCAGCGT |  |
| *zfAPLNR2b* | sense | CTGGGACTCACTGGGAATGG | 121 |
|  | antisense | GGGTCACCACAAAGGTCAGG |  |
| *zfAPLNR3a* | sense | ACAAGACACTCTCCGCAATG | 133 |
|  | antisense | TACAGCAGAGGGTTCAAACAG |  |
| *cActin* | sense | CCCAGACATCAGGGTGTGATG | 123 |
|  | antisense | GTTGGTGACAATACCGTGTTCAAT |  |
| *zfActin* | sense | CCGTGACATCAAGGAGAAGC | 196 |
|  | antisense | GATACCGCAAGATTCCATACC |  |

^a^All primers were synthesized by BGI (China).

^b^Restriction sites added in the 5’-end of the primers are underlined.
